# PORT: Generative EHR Pretraining for Pediatric Intraoperative Risk Prediction

**DOI:** 10.21203/rs.3.rs-10609619/v1

**Published:** 2026-08-12

**Authors:** Junseob Kim, Haoming Shi, Michael C. Fiedorek, Hannah D. Kilday, Henry P. Foote, Christoph P. Hornik, Rishikesan Kamaleswaran, Justin B. Long

**Affiliations:** 1 Department of Electrical and Computer Engineering, Duke University, Durham, NC, USA; 2 Department of Biomedical Engineering, Duke University, Durham, NC, USA; 3 Division of Pediatric Anesthesiology, Children’s Healthcare of Atlanta / Emory University School of Medicine, Atlanta, GA, USA; 4 Department of Pediatrics, Duke University Medical Center, Durham, NC, USA; 5 Duke Clinical Research Institute, Durham, NC, USA; 6 Department of Surgery, Duke University School of Medicine, Durham, NC, USA; 7 Department of Anesthesiology, Duke University School of Medicine, Durham, NC, USA

**Keywords:** intraoperative deterioration, pediatric perioperative care, electronic health records, generative pretraining, low-rank adaptation

## Abstract

Pediatric intraoperative deterioration (IoD) is a rare, high-consequence event with no validated pre-operative risk model. We developed PORT (Pediatric Operative Risk Transformer), a decoder-only transformer pretrained on perioperative electronic health record (EHR) timelines and adapted with low-rank adaptation, to predict IoD from pre-operative data. We studied a single-center retrospective cohort of 189,704 anesthesia encounters (127,874 patients, 2014–2021). On a patient-level held-out test set (37,948 encounters; 101 IoD events), PORT achieved an AUROC of 0.942 (95% CI 0.915–0.964) and an AUPRC of 0.237, outperforming a tuned BiLSTM and other baselines while remaining well calibrated (ECE 0.001); discrimination held in a temporally held-out post-2019 cohort (AUROC 0.949). Integrated Gradients attributed predictions to pre-operative tokens, and a high-risk subgroup (0.4% of encounters) captured 31.7% of IoD events. Lightweight adaptation of a pretrained generative EHR backbone thus yields accurate, calibrated, and interpretable risk estimates, supporting proactive perioperative planning.

## 1 Introduction

Perioperative complications remain a major source of morbidity and preventable mortality in pediatric surgery. Severe intraoperative events, including hemodynamic collapse, cardiac arrest, unplanned vasoactive escalation, and emergent invasive monitoring, are rare but high-consequence, with risk concentrated among medically complex children, particularly neonates and infants.^1,2^ For example, patients with congenital heart disease can experience intraoperative event rates as high as 5.2% during noncardiac procedures, with associated prolonged intensive care unit (ICU) stays and increased post-operative morbidity.^3,4^ Early preoperative identification of such high-risk patients could facilitate proactive perioperative planning, including specialist anesthesia coverage, extracorporeal membrane oxygenation (ECMO) standby, and targeted optimization strategies.

Contemporary risk-stratification tools fall into two classes. The American Society of Anesthesiologists (ASA) Physical Status Classification, the most widely used global pre-operative assessment, summarizes overall acuity in five ordinal levels but has limited inter-rater reliability and modest discrimination.^5^ Procedure-specific models, including the Society of Thoracic Surgeons Congenital Heart Surgery Database (STS-CHSD) mortality model^6,7^ and the Risk Adjustment for Congenital Heart Surgery (RACHS-1) complexity score,^8^ are concentrated in the cardiac-surgery subdomain and predict post-operative mortality from curated registry variables. For general pediatric surgery, the American College of Surgeons NSQIP-Pediatric program benchmarks risk-adjusted post-operative outcomes across pediatric surgical subspecialties from a curated variable set.^9^ Existing approaches thus remain dependent on manually curated features and coarse perioperative summaries.

Machine learning (ML) has been applied across the perioperative spectrum, but its application to pediatric surgery remains comparatively limited. In adult surgical populations, large-scale models have shown strong performance for post-operative complication and mortality prediction,^10,11^ and the Hypotension Prediction (HYPE) randomized trial showed that a real-time ML-derived index can reduce intraoperative hypotensive burden during elective noncardiac surgery.^12^ Within pediatric anesthesia, intraoperative ML predictors have emerged sporadically (e.g., for hypoxemia^13^), while the most sustained development has been in post-operative outcome prediction for high-risk subpopulations, notably cardiac surgery.^14–17^ These models typically rely on manually engineered features and produce single point estimates without quantifying predictive uncertainty.^18^ The key methodological challenge is therefore not the identification of additional risk factors, but the representation of existing clinical information. Recent learned-embedding approaches, such as the FLEX score,^19^ replace manually curated variable sets with attention-based representations derived from diagnostic and procedure codes; however, these models operate on code-set representations and do not explicitly model the temporal structure of the longitudinal pre-operative record.

Self-supervised, generative pretraining on electronic health record (EHR) sequences offers an alternative to task-specific supervised learning. Early work on Doctor AI^20^ used a recurrent neural network over longitudinal codes; Med-BERT^21^ and BEHRT^22^ pretrained contextual embeddings on tens of millions of patient records, while text-based models such as NYUTron^23^ and GatorTron^24^ leverage billions of words of clinical notes. A systematic review of 84 such models nevertheless found that most are evaluated on tasks disconnected from clinical deployment and few are validated outside their training domain.^25^ Enhanced Transformer for Health Outcome Simulation (ETHOS)^26^ and its successor ETHOS-ARES^27^ take a distinctive generative approach: a decoder-only transformer trained autoregressively on MIMIC-IV^28^ timelines in the Medical Event Data Standard (MEDS) format^29^ produces *N* future trajectories at inference, with outcome probabilities read off as the trajectory fraction containing the event. These approaches suggest a scalable framework for longitudinal perioperative risk modeling from raw EHR trajectories.

We introduce PORT (Pediatric Operative Risk Transformer), a decoder-only transformer pretrained autoregressively on MEDS timelines from Children’s Healthcare of Atlanta (CHOA) and adapted for intraoperative deterioration (IoD) prediction via Low-Rank Adaptation (LoRA).^30^ Our contributions are: (i) to our knowledge the first application of generative EHR pretraining to pediatric intraoperative risk prediction, yielding a reusable pretrained backbone re-adaptable to related perioperative outcomes via lightweight LoRA adapters, using code-identity-preserving tokenization over heterogeneous perioperative EHR sequences; (ii) a clinically grounded, reproducible pipeline for defining the five-component IoD outcome from structured events and free-text notes, transferable to other perioperative cohorts; (iii) calibrated per-encounter risk and uncertainty estimation without auxiliary calibration; and (iv) complementary interpretability analyses, including cohort-level category occlusion, to identify clinically salient predictors.

Our primary objective is to develop and evaluate PORT for pre-operative prediction of IoD in pediatric surgery using only information available before the patient enters the procedure suite. Evaluation focuses on three clinically relevant properties: discrimination, probability calibration, and predictive uncertainty. Secondary objectives are to characterize patient-specific drivers of risk via per-encounter attribution, identify which token categories contribute predictive signal at the cohort level through category occlusion, and quantify how much the model leverages longitudinal pre-operative context. Figure 1 summarizes the end-to-end pipeline together with a representative clinical decision-support view.

## 2 Results

### 2.1 Discrimination, calibration, and uncertainty

Table 1 summarizes model performance using the full pre-operative history. Among supervised baselines, the tuned bidirectional LSTM (BiLSTM) performed best (AUROC 0.906), with MEDS-aggregated models outperforming manual-feature models and ASA achieving an AUROC of 0.816. The three readouts on our pretrained backbone spanned a wide range of performance: zero-shot trajectory inference achieved an AUROC of 0.588, the linear probe 0.800, and PORT 0.942. The improvement over the tuned BiLSTM was statistically significant (ΔAUROC = +0.036, 95% CI [+0.011, +0.063], *p* = 0.006). PORT also degraded gracefully as predictions were generated further in advance of OR entry, with AUROC/AUPRC declining from 0.926/0.204 at 1 h to 0.790/0.012 at 12 h before OR entry (Fig. 2).

To evaluate robustness to temporal dataset shift, we partitioned the cohort chronologically (training on patients first seen before 2019 and testing on temporally held-out post-2019 patients) and re-trained PORT and all baselines from scratch. PORT demonstrated robust temporal generalizability, retaining excellent discrimination in the future cohort (AUROC, 0.949) while consistently outperforming all baseline models (BiLSTM, 0.877; XGBoost-MEDS, 0.847). The AUPRC decreased to 0.051, largely reflecting the substantially lower event prevalence (0.11% versus 0.27%), the reduced pre-operative history available for temporally new patients, and a modest loss of precision among the highest-confidence predictions. Importantly, both discrimination and calibration remained superior to those of all comparators, suggesting that the model generalizes well under temporal distribution shift. As expected for prospective implementation under changing prevalence, operating-point recalibration would be necessary prior to clinical deployment (Supplementary Section S5).

PORT produced well-calibrated risk estimates and quantified epistemic uncertainty through disagreement across three independently initialized models. Reliability analysis showed close agreement between predicted probabilities and observed IoD rates across risk deciles, whereas model uncertainty increased with predicted risk, indicating greater disagreement for higher-risk encounters (Fig. 3).

### 2.2 Per-encounter attribution analysis

Beyond aggregate discrimination, clinical use also requires explaining individual predictions in terms of specific tokens and timepoints. Figure 4 shows per-encounter Integrated Gradients (IG) attribution for four representative test encounters.

#### Case A ($\overset{ˆ}{p}=0.337$, IoD+).

A short pre-operative timeline with no single dominant driver: the risk-elevating attribution is distributed across a feeding tube, surgical-prophylaxis antibiotics (J01), a hospital admission, blood/fluid products (B05), and a sedative (N05), each modest (|IG|≤0.017). Consistent with distributed accumulation, no single token decisively drives the prediction.

#### Case B ($\overset{ˆ}{p}=0.619$, IoD+).

A high-acuity patient whose risk is dominated by active cardiovascular support: blood/fluid products (B05), an arterial line, a cardiac-stimulant/inotrope (C01), and vasopressin (H01) are the leading risk-elevating contributors, together with a recent pediatric-ICU episode. The high calibrated probability is anchored in these concrete markers of hemodynamic instability.

#### Case C ($\overset{ˆ}{p}=0.604$, IoD+).

A neonate managed in the neonatal intensive care unit. The elevated risk estimate is driven primarily by repeated cardiac stimulant (inotrope) administration, with Anatomical Therapeutic Chemical (ATC) class C01 medications providing the strongest positive contributions (|IG| up to 0.033; net medication IG ≈+0.17). Additional positive contributors include neonatal intensive care unit admission, a shock diagnosis (ICD-10 R57), and blood product exposure, whereas the presence of a peripheral intravenous catheter lowers the estimated risk. Overall, the dominant explanatory signals characterize an acutely ill, inotrope-supported neonatal cardiac patient rather than a stable elective case.

#### Case D ($\overset{ˆ}{p}=0.169$, IoD−).

A false positive driven by laboratory abnormalities rather than medications: point-of-care CO_2_, albumin, potassium, and temperature dominate the positive attribution (net laboratory IG ≈+0.18), while a recent pediatric ICU stay contributes negatively (no medications are present in the pre-operative window). The elevated score therefore reflects the patient’s abnormal peri-procedural physiology rather than acute intraoperative instability, illustrating a failure mode in which baseline derangement is decoupled from the eventual intraoperative course.

Beyond individual cases, token-level IG attribution correlated positively with each token’s empirical association with IoD across the vocabulary (Spearman *ρ* = 0.20, *p* = 7.6 × 10^−19^; Supplementary Fig. S9). Moreover, the top 20 attribution-ranked tokens were all more frequent in IoD+ encounters, indicating that IG concentrates on clinically meaningful risk-associated signals rather than spurious features. Together, these findings show that PORT grounds each prediction in clinically interpretable tokens spanning the longitudinal record, enabling case-level audit while revealing when elevated risk reflects baseline acuity rather than acute instability (Case D). Extended per-patient analyses spanning true positives, false positives, and true negatives are reported in Supplementary Section S6.

### 2.3 Category-level occlusion analysis

Complementing the encounter-level explanations, we quantified the population-level contribution of each token category using occlusion analysis. Per-category ΔAUROC and ΔAUPRC are summarized in Fig. 5 and Supplementary Table S13. Surgical Context tokens (procedure code, ASA score, admission type, patient class, and operative-window markers) produced the largest reduction in discrimination when masked (ΔAUROC = −0.056, ΔAUPRC = −0.029), identifying pre-operative surgical and anesthetic context as the dominant source of predictive information. Surgical History (PROCEDURE; ΔAUROC = −0.045), ICD-10 diagnoses (ΔAUROC = −0.020), and Medications (ATC; ΔAUROC = −0.014) were the next most influential contributors to AUROC. In contrast, Medications had by far the largest impact on precision–recall performance (ΔAUPRC = −0.189), reducing AUPRC from 0.237 to 0.048 when masked. This disparity likely reflects PORT’s code-identity-preserving dose encoding, which captures clinically meaningful treatment intensity, particularly pre-operative vasopressor and inotrope use. Among the remaining categories, Language, Point of Origin, and Surgical History made modest contributions to AUPRC (all ΔAUPRC ≥ −0.008), whereas laboratory values, vital signs, anesthesia events, and free-text history individually had negligible effects (all |ΔAUROC| ≤ 0.001).

The category ranking is consistent with clinical practice at OR entry, where current physiologic status and active perioperative management are generally more informative than historical comorbidity burden. The limited incremental contribution of laboratory variables likely reflects redundancy with vital signs and medication-related features, as abnormal pre-operative laboratory findings are typically reviewed and addressed before elective procedures proceed.^31^ The contribution of Point of Origin and Language further suggests that contextual patient characteristics, including healthcare utilization patterns, may provide information beyond routinely captured clinical variables alone.

### 2.4 Risk stratification and clinical utility

Figure 6 shows how predicted risks translate into clinically meaningful risk stratification. Ranking test encounters by predicted risk, PORT concentrated IoD events more efficiently than every baseline. Flagging the highest-risk 10% of encounters captured 83.2% of all IoD events, compared with 72.4% for the next-best BiLSTM and ≤61% for the tabular and clinical models. This advantage persisted across the clinically relevant flagging range; among the highest-risk 25% of encounters, PORT captured an additional 12% of all IoD events relative to the BiLSTM. A high-risk subgroup defined by predicted risk > 10% comprised only 0.4% of encounters yet captured 31.7% of all IoD events, and at the highest predicted-risk level (> 60%), 12 of 14 encounters (86%) were IoD-positive. Table 2 summarizes performance across representative operating points, demonstrating favorable trade-offs among sensitivity, specificity, and PPV while maintaining an NPV above 0.99 throughout.

Performance remained robust across clinically relevant subgroups. Discrimination was stable across ASA-defined strata despite substantial differences in IoD prevalence, and decision-curve analysis demonstrated consistently greater net benefit than all comparator models (Supplementary Fig. S12). PORT also retained discrimination comparable to that of the full cohort in the noncardiac surgery subgroup while outperforming all comparator models (Supplementary Table S2). In contrast, discrimination remained limited for all models in cardiac surgery, where the higher AUPRC largely reflected increased event prevalence rather than improved ranking performance.

## 3 Discussion

PORT combines generative autoregressive EHR pretraining with parameter-efficient LoRA adaptation for pre-operative prediction of pediatric intraoperative deterioration. Although it achieved the strongest discrimination among all evaluated models (AUROC 0.942, AUPRC 0.237) while remaining well calibrated (ECE 0.001), its principal contribution extends beyond predictive performance. By combining calibrated risk estimation with patient-level interpretability, predictive uncertainty, and a reusable generative backbone, PORT establishes a foundation model that can be efficiently adapted to a broad range of perioperative prediction tasks.

Supervised adaptation was necessary because zero-shot trajectory inference is ill suited to partially unstructured composite outcomes. The standard ETHOS readout estimates outcome probability from the fraction of generated trajectories containing a target token, an assumption that breaks down when the target is only partially represented in the vocabulary. Accordingly, zero-shot inference performed poorly for IoD (AUROC 0.588), whose definition includes heterogeneous structured events and a free-text-derived component excluded from tokenization. The strong performance of a linear probe on the frozen backbone (Table 1) indicates that this limitation arises from the readout rather than the representation itself, while lightweight LoRA adaptation efficiently converts the pretrained representation into an accurate task-specific predictor without sacrificing predictive uncertainty estimation.

Existing pre-operative pediatric risk assessment relies primarily on ASA physical status^5^ and procedure-specific cardiac scores such as RACHS-1 and STS-CHSD.^6–8^ The major pre-operative determinants of pediatric IoD, including high ASA status, young age, emergent surgery, and congenital heart disease, are already well established.^32–34^ PORT therefore does not seek to rediscover these factors; rather than relying on a fixed, manually curated variable set, it integrates heterogeneous longitudinal perioperative information directly from raw tokenized timelines encompassing care trajectory, surgical history, medications, procedures, laboratory values, vital signs, and device exposure. Surgery-type–stratified analyses (Supplementary Table S2) suggest that this advantage arises primarily in the much larger noncardiac population, where PORT retained discrimination comparable to that of the full cohort and consistently outperformed competing models. In contrast, discrimination remained limited for all models in the uniformly high-acuity cardiac-surgery subgroup, likely because the pre-operative signals emphasized by PORT, particularly medications, care trajectory, and surgical history, are nearly ubiquitous in congenital cardiac patients, whereas much of the remaining risk is procedure-specific or intraoperative. Dedicated procedure-specific scores such as RACHS-1 and STS-EACTS therefore remain more appropriate in that setting.

Compared with FLEX,^19^ which applies learned EHR embeddings to adult postoperative risk prediction using static set-based representations, PORT employs sequential generative modeling for pediatric intraoperative risk prediction. More broadly, prior generative EHR pretraining studies were conducted on large-scale datasets,^21,22,26,35–37^ whereas PORT was developed on a comparatively small pediatric surgical cohort with sparse rare-event labels. ETHOS-style autoregressive pretraining^26^ nevertheless produced well-calibrated risk estimates without post hoc recalibration, and LoRA^30^ efficiently incorporated task-specific supervision, indicating that generative EHR pretraining remains effective in specialized pediatric perioperative settings with limited data and rare outcomes.

The attribution analyses indicate that PORT relies primarily on clinically plausible markers of perioperative vulnerability rather than on idiosyncratic correlations. Across the full cohort (Supplementary Fig. S9), the most highly attributed tokens were predominantly prior cardiac surgical-history markers, including established congenital cardiac procedures such as Fontan, Glenn, and arterial-switch operations as well as ECMO cannulation, reflecting information contained in the longitudinal record rather than in the index procedure itself. Consistent with the cohort-wide concordance between attribution and association reported in the Results, this supports the interpretation that attribution highlights genuinely risk-associated signals rather than spurious features. Because repeated chronic-disease and prior-event markers can introduce optimism in EHR foundation models,^38^ we additionally evaluated encounters with no prior IoD history and observed essentially unchanged performance (AUROC 0.940, AUPRC 0.247 vs. 0.942/0.237 in the full cohort; Supplementary Table S9), indicating that PORT is not primarily identifying patients who have previously deteriorated.

Clinically, PORT is best suited for deployment within higher-acuity subgroups, as the low population incidence of IoD (0.27%) limits PPV even for highly discriminative models, whereas enrichment of event prevalence yields a more clinically actionable operating point. This deployment strategy is supported by stable discrimination across ASA strata, the applicability of population-derived operating thresholds within subgroups, and positive net benefit across clinically relevant decision thresholds. Deployment also appears feasible: inference required only 7.2 ms and 318 MB of GPU memory (Supplementary Table S16), while most predictive information was retained using only a seven-day pre-operative history (Supplementary Section S4) and when predictions were generated 1 h before OR entry (AUROC 0.926, AUPRC 0.204; Fig. 2c). Such lead time could facilitate earlier staffing decisions (e.g., pediatric cardiac anesthesia or intensivist coverage), escalation of monitoring (e.g., early invasive monitoring or transesophageal echocardiography), and preparation of contingency resources (e.g., ECMO, rapid-response teams, and blood products), consistent with pediatric anesthesia safety initiatives emphasizing early recognition, team readiness, and escalation pathways.^39–41^ Realizing earlier prediction in practice, however, will require models specifically trained and validated for that lead time.

Several limitations should be noted. The backbone was trained exclusively on pediatric surgical data; although initialization from a larger MIMIC-IV–pretrained model could improve rare-event detection, it would require harmonization of heterogeneous clinical vocabularies. Consistent with this, data-efficiency analyses (Supplementary Section S3) showed that PORT consistently outperformed the BiLSTM only when at least 50% of the training data were available, suggesting that simpler sequence models may remain competitive in substantially smaller cohorts. IoD2 labels were derived from keyword matching, LLM-assisted screening, and independent adjudication by two pediatric anesthesiologists, but residual label noise cannot be excluded, and free-text inputs were omitted because they are unavailable or impractical before OR entry. IG provides local explanations rather than causal inference, and attribution estimates depend on the choice of baseline embedding and integration discretization. This retrospective single-center study did not include geographic external validation or prospective evaluation, and practical deployment depends on reliable pre-operative availability of OR-entry timestamps, ASA status, and procedure codes to prevent information leakage. Although performance was stable across ASA-acuity and sociodemographic strata, algorithmic fairness was not formally assessed or mitigated and warrants dedicated evaluation before deployment. Finally, alternative parameter-efficient adaptation strategies were not evaluated exhaustively, and potential optimism from recurrent chronic-condition or prior-deterioration tokens warrants further scrutiny despite stable sensitivity analyses.

Future work should extend the same tokenization and generative modeling framework beyond the pre-operative period to longitudinal perioperative forecasting, incorporating additional modalities such as clinical notes, physiologic waveforms, and device telemetry so that risk estimation can evolve dynamically throughout the perioperative course. A second priority is to establish the reusability of the pretrained backbone by training task-specific LoRA adapters for related outcomes such as PACU deterioration, escalation of care, and 30-day readmission. Prospective and external validation remain essential prerequisites for clinical deployment.

Pre-operative IoD risk in pediatric surgery has historically lacked models that are simultaneously discriminative, well-calibrated, uncertainty-aware, and per-encounter interpretable. PORT addresses these requirements within a single generative EHR framework, achieving clinically meaningful risk stratification together with calibrated uncertainty estimates and patient-level attribution onto named perioperative tokens. More broadly, generative autoregressive EHR pretraining paired with lightweight LoRA adaptation can recover clinically meaningful signal even for rare outcomes partially defined through unstructured data. Because the same backbone can be re-adapted to related outcomes, the framework provides a scalable foundation for longitudinal perioperative risk modeling across multiple surgical endpoints.

## 4 Methods

### 4.1 Cohort, outcome, and tokenization

#### Study cohort and data sources.

We used retrospective EHR data from the Egleston and Scottish Rite campuses of Children’s Healthcare of Atlanta (CHOA). Anesthesia encounters recorded in the anesthesia information management system (AIMS) spanned April 2014 to November 2021, while structured longitudinal EHR data were available from March 2007 through October 2022, providing up to 15.5 years of clinical history. The study population comprised all AIMS-recorded anesthesia encounters during the study period. We excluded 20,570 encounters (9.78%) lacking a documented operating-room entry timestamp, as these primarily reflected out-of-operating-room procedural anesthesia (e.g., cardiac catheterization, interventional radiology, imaging, and sedation) rather than operative surgical care. The final cohort included 189,704 anesthesia encounters from 127,874 patients (Supplementary Fig. S10); patient and encounter characteristics are summarized in Table 3. We harmonized 12 structured EHR tables into the MEDS format,^29^ yielding 26,504,708 clinical events across 53,705 unique codes; data-source coverage within the analysis cohort is summarized in Supplementary Table S5.

#### IoD label definition.

IoD was defined as a composite of five intraprocedure events selected by pediatric anesthesiologists to capture clinically significant intraoperative deterioration requiring active intervention, rather than any single mechanism of harm. Four components were derived from structured EHR data (anesthesia events, medications, and lines/drains), and one from free-text annotations in the AIMS Quick Note field. Component definitions, including dose thresholds, timing rules, and exclusion criteria, were iteratively refined with clinical input to distinguish acute deterioration from routine management. The label window spanned the intraprocedure period from anesthesia start to OR exit, while model inputs were restricted to pre-operative data available at the In OR timestamp. Two of the composite components are defined by the clinical response to deterioration because acute intraoperative instability is frequently documented only through the intervention it provokes rather than as a discrete structured measurement; IoD3 and IoD4 therefore serve as objective, timestamped surrogates for a deterioration event. The accompanying dose thresholds, timing constraints, and exclusion rules were calibrated to separate genuine rescue from protocolized or prophylactic management, so that an intervention contributes to the label only when its context indicates acute escalation. Because model inputs were truncated at the OR-entry timestamp, the pre-operative medication tokens used as predictors are temporally and mechanistically distinct from the intraoperative vasoactive administrations that define the outcome, precluding label leakage or definitional circularity.

The five components were: cardiac arrest requiring cardiopulmonary resuscitation (CPR; IoD1), Quick Note keyword deterioration (IoD2), IV vasoactive bolus (IoD3), vasoactive infusion escalation (IoD4), and emergent arterial line placement (IoD5) (Table 4). For IoD2, candidate events were identified using keyword matching against a 31-term list, followed by regex exclusion, a screen using an ensemble of three large language models (LLMs), and final independent adjudication of the remaining candidates by two pediatric anesthesiologists (Table 5). Operational definitions for IoD2–IoD5 are detailed in the IoD outcome components subsection below.

#### MEDS tokenization.

A flat-code MEDS vocabulary derived directly from the source tables comprised 47,691 tokens, of which approximately 67% occurred in fewer than ten training events. To construct a compact medical vocabulary aligned with the ETHOS framework, we applied four transformations (detailed in the Tokenization details subsection below). We hierarchically decomposed ICD-10-CM and ATC codes and resolved drug-to-ATC mappings through an LLM-assisted pipeline with manual review. We then applied frequency-based long-tail truncation with per-category OTHER fallbacks and decomposed demographic and socioeconomic fields atomically. As an example of this truncation, the free-text primary procedure name recorded at OR entry, a model input, was retained as its 500 most frequent values plus an OTHER bin rather than mapped to a standardized code such as Current Procedural Terminology [CPT]. The resulting vocabulary contained 6,170 tokens (Supplementary Table S6), consistent with recent systematic evaluations favoring joint code–value event tokens and compact vocabularies for EHR transformer pretraining.^42,43^

All codes carrying numeric values (laboratory results, vital signs, medication doses, ASA score, gestational age) were tokenized as code–quantile pairs [code*, Q*_*k*_], where *Q*_*k*_ is a shared decile token computed from per-code training-set boundaries. Inter-event temporal gaps were discretized into 19 quasi-logarithmic interval tokens spanning 5 minutes to 6 months, and three sentinel tokens (TIMELINE_END, OUTCOME//DEATH, MED//UNMAPPED) controlled trajectory generation. Median patient timeline length was 112 tokens (p95: 1,199); the longest 3.2% exceeded the 2,048-token context window and were truncated to the most recent 2,048 tokens. A worked tokenization example is shown in Supplementary Table S7.

#### Dataset splits.

Patients were split into training (70%), validation (10%), and test (20%) by patient-level random assignment stratified on whether the patient had any IoD+ encounter, yielding 132,671 / 19,085 / 37,948 encounters across 89,536 / 12,796 / 25,542 patients (Supplementary Table S8); all of a patient’s encounters stayed in one split to prevent leakage. Each encounter was treated as an independent sample, reflecting the clinical reality that risk assessment is performed separately for each surgical episode. Consequently, later encounters could incorporate information from a patient’s prior surgeries (47.3% of test encounters), mirroring the information available during routine pre-operative evaluation. Prior IoD+ events were rare (133 test encounters, 0.4%), and sensitivity analyses restricted to first encounters and to encounters without prior IoD are reported in Supplementary Table S9. Median available pre-operative history was 291 days (interquartile range [IQR] 3–1,414), with 27.7% of encounters having under 7 days (Supplementary Fig. S11).

### 4.2 Tokenization details

#### Diagnosis codes.

ICD-10-CM codes from the medical-history, problem-list, and cardiology-encounter tables were decomposed into up to three hierarchical sub-tokens by slicing the dot-stripped code at positions [0:3], [3:6], and [6:], following the ETHOS tokenizer,^26^ compressing 34,322 flat ICD tokens to 3,216 hierarchical tokens. A shared level-1 token across related diagnoses (e.g., all Q21. *x* congenital septal defects) encourages representation sharing, while deeper tokens retain the granularity to separate clinically distinct subtypes.

#### Medications: LLM-assisted ATC mapping.

The ATC ontology’s five levels (anatomical group to chemical substance) suit token sharing, but the MIMIC-IV–derived drug-to-ATC dictionary shipped with ETHOS^26^ covers only a fraction of pediatric drugs. We mapped the 10.5M medication events in three stages: normalizing route, formulation, and dose suffixes collapsed 2,690 raw strings to 2,493 canonical names; exact dictionary lookup resolved 1,731 of these (69.4% of names, 67.3% of events); and the residual 762 drugs were classified by zero-shot hierarchical prompting of Llama 3.3 70B Instruct,^44^ using dose-informed prompts,^45^ reaching 78% pilot accuracy at ATC level 3. We manually reviewed all 762 mappings (686 accepted, 67 corrected, 9 flagged) and decomposed each finalized code into three hierarchical tokens, yielding a 277-token medication vocabulary.

#### Long-tail tables.

For the heavy-tailed laboratory, surgical-history, structured-data, and free-text history tables, we retained the most frequent codes covering ≥95% of training events (per-category cutoffs in Supplementary Table S6) and mapped the remainder to category-specific OTHER tokens; codes below this cutoff occurred fewer than ten times and were mostly noise. Per-category OTHER tokens preserve a learnable “rare event from this table” signal without inflating the vocabulary.

#### Anesthesia patients table.

This table contributed several token streams. Surgical-context tokens encoded the primary procedure (top-500 + OTHER), ASA status, admission type, patient class, and operative-window markers (520 tokens); the ENCOUNTER//AN//OR_ENTRY token both delimits the prediction time and serves as the readout position for the PORT classification head. Eight pre-procedure vital signs were paired with per-code quantile tokens, and demographics were decomposed atomically into race (8), sex (2), and ethnicity (4) to share representation across patients differing in a single attribute. Four socioeconomic fields—primary language, insurance, point of origin, and home county (top-*k* + OTHER; 6, 6, 6, and 51 tokens)—were added under the hypothesis that referral pathway, payer mix, and language modulate baseline acuity in this safety-net population.

#### Anesthesia events and care-trajectory tables.

Anesthesia events were collapsed into 12 atomic tokens; AN_EVENT//QUICK_NOTE records only a note’s existence and timestamp (its text is used to derive the IoD2 label but is not tokenized), and AN_EVENT//CPR doubles as the IoD1 stop token during zero-shot inference (Supplementary Table S11). Care-trajectory tables used paired enter/exit tokens: ADT transfers across 54 locations (106 tokens) and lines, drains, and airways across 40 device types (80 place/remove tokens, including the arterial-line tokens underlying IoD5). Transfusions were collapsed to 14 product-type tokens without separate dose tokens, since transfused volumes overlapped heavily across products. Free-text cardiology notes were excluded, as ETHOS consumes only structured event tokens.

### 4.3 IoD outcome components

#### IoD2: deterioration keywords in free-text Quick Notes.

The anesthesia information management system records a free-text “Quick Note” field used intraoperatively to flag clinically significant events not captured in structured fields. Candidate IoD2 events were identified by case-insensitive substring matching of Quick Notes against a 31-concept keyword list developed with a board-certified pediatric anesthesiologist, covering five concept groups: (i) cardiopulmonary arrest and resuscitation (*chest compression, CPR, code, PALS, arrest, defib, defibrillation, DCCV, cardioversion, ECMO, pulseless, no pulse*); (ii) hemodynamic deterioration (*deteriorated, deterioration, hypotension, shock, brady, cardiac output*); (iii) vasoactive medication mentions (*epinephrine, epi, dopamine, phenylephrine, norepi, norepinephrine*); (iv) arrhythmias (*arrhythmia, SVT, Vtach / v-tach / ventricular tachycardia, vfib / v-fib / ventricular fibrillation, fibrillation*); and (v) emergency activation (*emergently, help, anesthesia now, overhead, echo*). This broad match yielded 6,535 notes from 3,502 distinct anesthesia encounters, which were refined through a staged cascade—regex context exclusion, an LLM-ensemble rescue-versus-managed screen, and independent clinician adjudication—with step counts and category breakdowns in Table 5.

#### Step 1: regex-based context exclusion.

We first removed keyword false positives with rule-based patterns targeting four recurrent contexts: “epi” as a substring of *epidural*, ophthalmic phenylephrine eye drops, “echo” in echocardiography-scheduling notes, and deliberately induced (planned) hypotension. Step 1 reduced the pool from 6,535 to 3,805 notes and from 3,502 to 1,785 encounters (per-context counts in Table 5).

#### Step 2: LLM-ensemble rescue-versus-managed screen.

The surviving candidates contained many context-dependent false positives beyond rule-based reach. Each candidate encounter, with its flagged Quick Note text, was independently classified as KEEP (a genuine acute intraoperative deterioration requiring active rescue) or EXCLUDE (a false-positive keyword match or a planned/protocolized event) by an ensemble of three open-weight large language models from distinct families—Qwen2.5–72B-Instruct,^46^ MedGemma-27B,^47^ and DeepSeek-R1-Distill-Llama-70B^48^—each prompted as a pediatric anesthesiologist under a structured rescue-versus-managed rubric with deterministic decoding (temperature 0). Models defaulted to KEEP under uncertainty to preserve sensitivity, and encounters were retained on a majority (≥2 of 3) KEEP vote, yielding 284 candidates for the Step-3 few-shot re-screen and clinician adjudication. The rubric is summarized below.

#### Step 3: few-shot re-screen and clinician adjudication.

The 284 candidates were re-screened by the same ensemble, augmented with the operative procedure, a cardiac-surgery context guide (planned or protocolized cardiac events that do not constitute rescue), and 18 clinician-adjudicated few-shot examples, reducing the pool to 159. Two pediatric anesthesiologists then independently adjudicated these 159 encounters, each given the operation and asked whether the event represented genuine unplanned intraoperative deterioration (Cohen’s *κ* = 0.58). Reviewer agreement, joint resolution of the disagreements, and a targeted re-screen of previously excluded cardiac encounters yielded the final adjudicated IoD2 label of 91 encounters and a composite IoD label of 449 analysis-cohort encounters. Only the assignment of positive labels was revised during adjudication; the underlying rescue-versus-managed definition remained fixed.

#### LLM-ensemble classification rubric.

Each model was told the operation and shown the note(s), then asked whether the patient required *rescue* or whether the event was *managed* within the routine course of the operation—severity of wording explicitly not being the criterion. A note was labeled KEEP only for (a) actual resuscitation (chest compressions, CPR, cardiac massage for arrest, asystole, PEA, “no pulse,” or defibrillation/cardioversion for a cardiac arrest) or (b) failure of routine management (unplanned ECMO or mechanical circulatory support, chest reopening or return to cardiopulmonary bypass for instability, an operation aborted or postponed for instability, or instability refractory to maximal therapy). All other events were EXCLUDE, even when dramatically worded: hypotension, bradycardia, or arrhythmia responding to routine treatment; airway events not progressing to chest compressions; fluid boluses or infusion changes; and documentation, transport, handoff, imaging-logistics, equipment-artifact, topical or local epinephrine, or planned defibrillation-threshold tests. For congenital cardiac surgery, a context caveat specified that ventricular fibrillation, defibrillation or internal cardioversion, open-chest cardiac massage, planned deep-hypothermic circulatory arrest, ECMO-flow adjustments, surgeon-administered intracardiac epinephrine, and pressor-treated hypotension are part of the routine (usually on-bypass) course and are not rescue. Each model returned a single JSON decision with fields label, trigger, and a brief reason.

#### IoD3: IV vasoactive bolus.

IoD3 was identified from the Medication Administration table by matching intraprocedure IV administrations of epinephrine, phenylephrine, ephedrine, vasopressin, adenosine, and atropine. The dose thresholds embed clinical context: phenylephrine and ephedrine require more than two bolus doses to exclude single prophylactic doses commonly given at induction, and atropine is restricted to patients older than 3 months because atropine pre-medication is routine in neonates and would otherwise dominate the label.

#### IoD4: vasoactive infusion escalation.

IoD4 required either a new infusion started after anesthesia start or a more-than-100% rate increase during the intraprocedure window for dopamine, epinephrine, milrinone, norepinephrine, phenylephrine, or vasopressin. The doubling threshold is the conventional anesthetic definition of meaningful pharmacologic escalation rather than dose titration.

#### IoD5: emergent arterial line placement.

IoD5 required a new arterial-line LDA entry timed after the procedure start time, distinguishing emergent intra-incision vascular access (a hemodynamic-instability marker) from routine pre-incision arterial access placed for monitoring.

### 4.4 PORT model and baselines

#### Backbone architecture and pretraining.

The PORT backbone is a decoder-only causal language model in the GPT-2 family,^49^ trained autoregressively with cross-entropy loss to predict the next event token in a patient’s MEDS timeline under causal attention masking. This choice follows ETHOS, which showed that generative trajectory sampling yields calibrated outcome probabilities for adult inpatients without task-specific supervision, and suits left-to-right clinical timelines. We trained from random initialization, as our pediatric-surgical code distribution overlaps little with adult corpora. Optimization used AdamW with a cosine schedule for 200,000 steps on 8 NVIDIA H200 GPUs (≈17 h; full hyperparameters in Supplementary Table S10).

#### Zero-shot IoD inference.

For each test encounter, we constructed a conditioning prefix from all tokens with timestamp ≤ In OR and sampled *N* = 100 trajectories autoregressively at temperature 1.0. Each trajectory was terminated at an IoD+ stop token (Supplementary Table S11), the DEATH or TIMELINE_END sentinel, or 24 h of cumulative simulated time; the DEATH sentinel only marks timeline end and was not itself counted as an IoD event, as intraoperative death is rare and preceded by the CPR signal (IoD1). The IoD probability ${\overset{ˆ}{p}}_{\text{IoD}}$ was the fraction of trajectories containing an IoD+ stop token; predictive uncertainty was the standard deviation of this Bernoulli indicator across trajectories.

#### Supervised adaptation: linear probe and LoRA.

The pretrained backbone alone is insufficient for IoD prediction because IoD2 derives from free-text Quick Notes excluded from tokenization. Supervised adaptation is therefore required, but full fine-tuning is undesirable given the extreme class imbalance and the advantage of distributing lightweight task-specific updates over a shared backbone.

We therefore evaluated two parameter-efficient adaptation strategies sharing the same classification head, a 2-layer multilayer perceptron mapping the hidden state at the ENCOUNTER//AN//OR_ENTRY position to the IoD probability. The first is a linear probe that freezes the pretrained backbone and trains only the head, isolating the pretrained representation’s contribution. Our primary method, PORT, additionally inserts trainable LoRA^30^ adapters into the query-key-value (QKV) projections of all six attention layers while keeping backbone weights frozen. For each projection matrix $W\in R^{3d\times d}$,

(1) $$W^{\prime }=W+\frac{\alpha }{r}BA,\;A\in R^{r\times d},\;B\in R^{3d\times r},$$

where *r* denotes the adapter rank and *α* scales the adapter contribution. Only the low-rank matrices **A** and **B** are trainable; restricting adapters to the QKV projections lets attention re-weighting carry the task-specific signal efficiently. Both variants were trained using unweighted binary cross-entropy (BCE):

(2) $$ℒ(\phi )=-\frac{1}{\left|𝒟\right|}\sum_{i}\left[y_{i}\;\text{log}\;{\overset{ˆ}{y}}_{i}+\left(1-y_{i}\right)\text{log}\left(1-{\overset{ˆ}{y}}_{i}\right)\right].$$

A 5 × 4 sweep over adapter rank (*r* ∈ {4, 8, 16, 32, 64}, *α* = 2*r*) and training objective (weighted BCE, unweighted BCE, focal loss, or 5× positive-class oversampling; Supplementary Section S2) selected unweighted BCE with *r* = 8. Including the classification head, the final model trained just 0.73% of backbone parameters (Supplementary Table S10) and was repeated across three random seeds.

#### Baseline models.

PORT was compared against baseline families designed to isolate the incremental contribution of model complexity. First, we evaluated logistic regression using only the ASA physical status score drawn from the pre-operative anesthesia note in its last documented form at or before OR entry,^5^ the standard pre-operative risk assessment in anesthesia practice. Second, we trained conventional tabular models (LR and XGBoost) on 61 structured features routinely available at OR entry, including demographics, ASA, admission type, patient class, pre-procedure vital signs, and one-hot encoded indicators for the 50 most common procedures (> 80% of cases). Third, we extended these models with 84 additional MEDS-derived EHR summary features, including recent laboratory values, medication counts, ICD-10 chapter indicators, prior hospitalization counts, ICU/device flags, and transfusion counts, for a total of 145 inputs. Finally, we trained a BiLSTM^50^ directly on raw MEDS event sequences, providing a strong sequence-modeling baseline for evaluating generative pretraining. All baselines used unweighted losses matching PORT, with hyperparameters tuned separately and the highest-AUPRC configuration selected (Supplementary Table S12).

### 4.5 Evaluation and interpretability analyses

#### Metrics and calibration.

We report AUROC, AUPRC, the Brier score, and the Brier Skill Score (BSS). Because AUROC can be insensitive to severe class imbalance, AUPRC served as the primary discrimination metric for this low-prevalence outcome.^51–53^ Calibration was assessed using the Brier score, BSS (1 − Brier_model_/Brier_ref_) relative to a constant-prevalence reference, 10-bin reliability diagrams, and expected calibration error (ECE).

Subgroup analyses stratified performance by ASA class to assess robustness across the perioperative acuity spectrum. Performance was also evaluated separately for congenital cardiac and noncardiac surgery. Cardiac surgery was defined by the operating surgeon (a clinician-curated roster of the institution’s congenital cardiac surgeons) and verified using an anatomic procedure-name classification based on established congenital heart surgery nomenclature (Supplementary Section S1).

#### Cohort-level category occlusion.

We quantified each token category’s marginal contribution by masking, one category at a time, all tokens in one of 18 clinical vocabulary categories (Supplementary Table S6) together with their adjacent quantile tokens for numeric events, and re-evaluating PORT on the full test set. Each category’s ΔAUROC and ΔAUPRC against the unmasked baseline quantify its cohort-level contribution.

#### Per-encounter Integrated Gradients attribution.

For per-encounter interpretability, we used IG,^54^ which attributes model predictions to individual input tokens by integrating gradients along a straight-line path from a baseline embedding x′ to the observed embedding x:

(3) $${\text{IG}}_{i}(\text{x})=\left(x_{i}-x_{i}^{\prime }\right)\int_{\alpha =0}^{1}\frac{\partial F\left({\text{x}}^{\prime }+\alpha \left(\text{x}-{\text{x}}^{\prime }\right)\right)}{\partial x_{i}}d\alpha ,$$

where *F* denotes the sigmoid-mapped model output at the OR-entry hidden state. We used the all-zero (PAD) embedding as baseline and approximated the integral with a 50-step Riemann sum. Attributions were summed across hidden dimensions and aggregated at the token or vocabulary-category level. By the completeness property of IG, the summed attribution equals *F* (x) − *F* (x′), a self-consistency check. Full-context IG was computed for all 37,948 test encounters.

#### Context-window ablation.

We characterized how much pre-operative history PORT meaningfully leverages by re-training and re-evaluating PORT (and all baselines) with look-back windows of 7, 30, 90, and 365 days plus the patient’s complete history (Supplementary Section S4); the prediction point and 24 h generation horizon were held fixed across windows. Conversely, to assess how early a prediction could be issued, we evaluated the trained PORT at a range of lead times before OR entry (15 min to 7 days) by masking tokens timestamped within the final interval before OR entry and reading the OR-entry hidden state; since PORT was trained for OR-entry readout, these values are a conservative lower bound.

## Supplementary Material

1

## Figures and Tables

**Figure 1:**
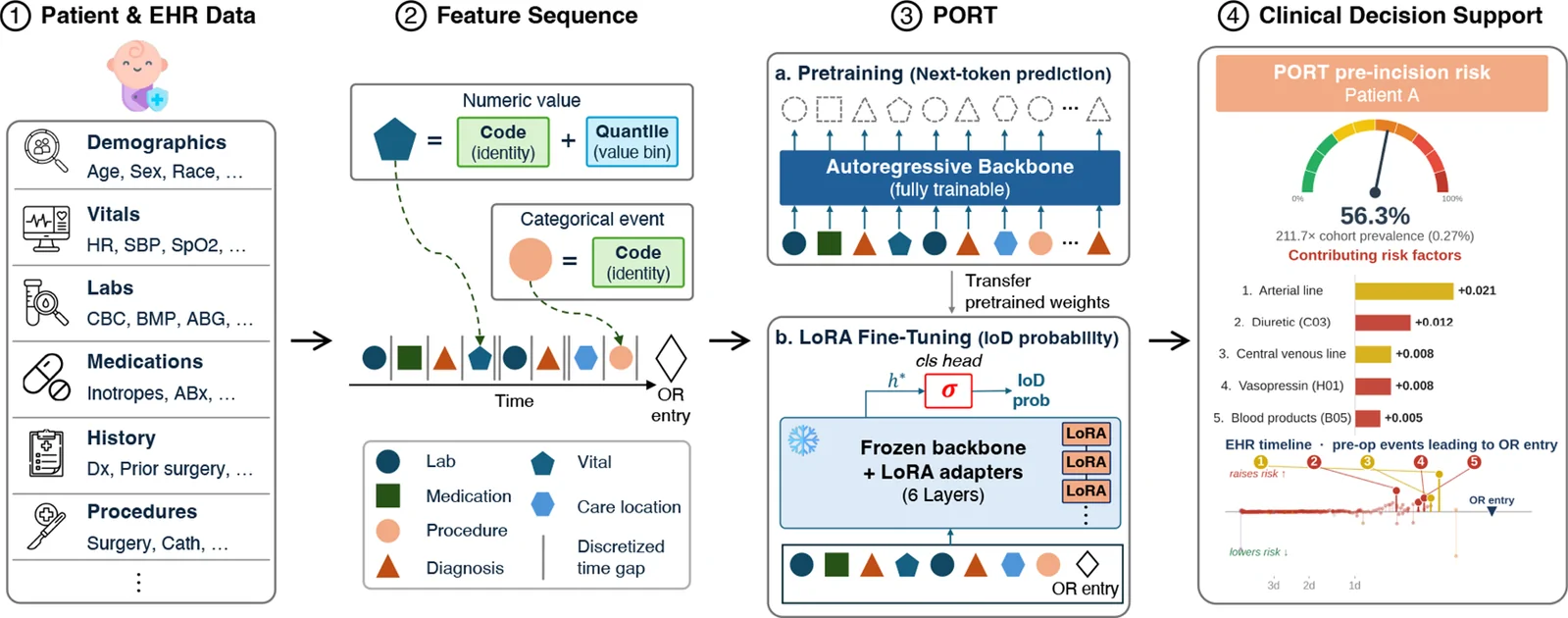
Overview of the PORT pipeline. (1) Heterogeneous pediatric perioperative EHR records. (2) Chronological tokenization into code, quantized value, and time-gap tokens (diamond: OR entry). (3) A six-layer decoder-only transformer pretrained on the 6,170-token MEDS vocabulary is adapted for IoD prediction with LoRA on the attention projections; the frozen backbone outputs the OR-entry hidden state h* for classification. (4) Dashboard displaying predicted IoD probability, top-five Integrated Gradients drivers, and the pre-operative EHR timeline.

**Figure 2:**
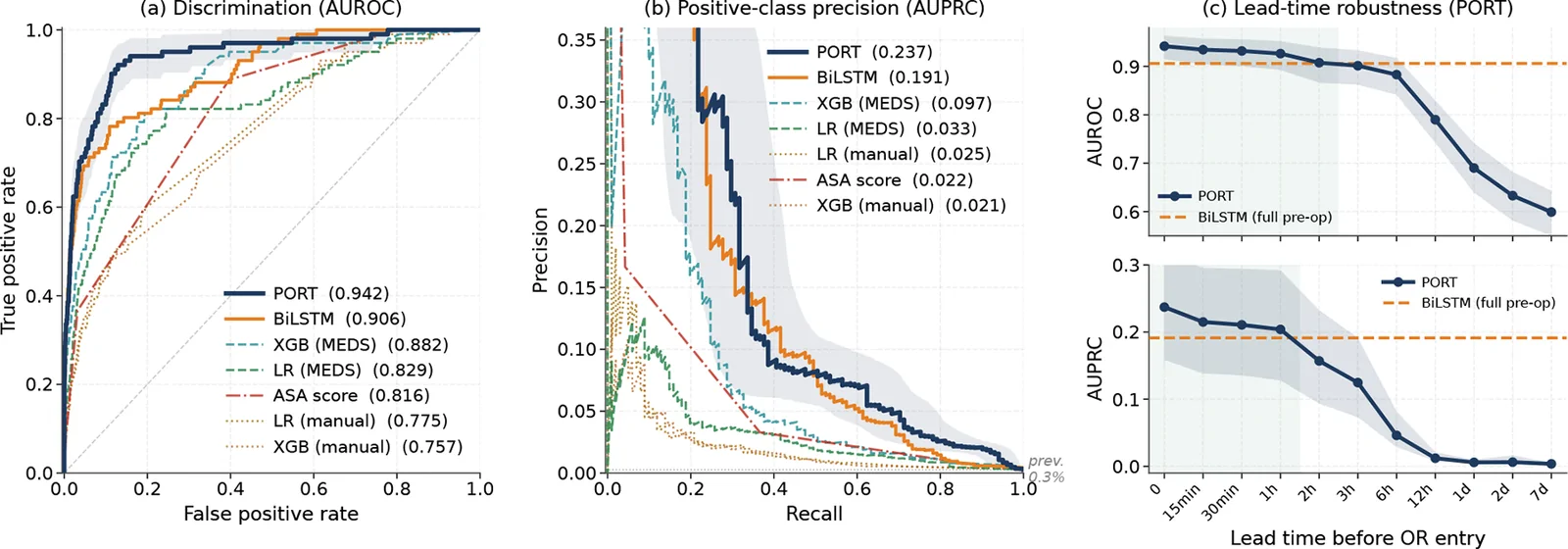
Test-set discrimination (*n*=37,948). (a) ROC and (b) precision–recall curves, with legends ordered by the corresponding metric. The shaded band denotes the 95% bootstrap confidence interval for PORT (navy); confidence intervals for all models are reported in Table 1. The random-classifier baseline in the PR plot (0.003) equals the 0.27% outcome prevalence. (c) PORT performance versus prediction lead time before OR entry, obtained by masking tokens newer than each cutoff. The navy band shows the patient-clustered bootstrap 95% confidence interval, and the dashed line the full-history BiLSTM baseline. Green shading indicates the lead-time range over which PORT outperforms this baseline.

**Figure 3:**
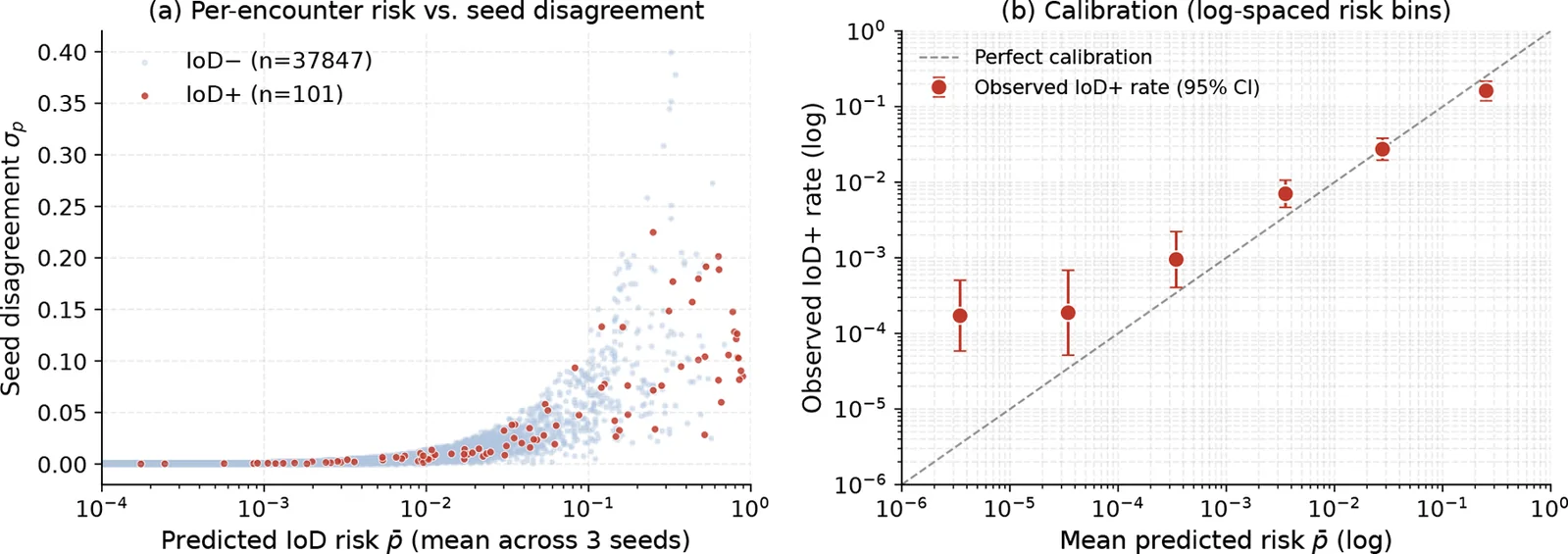
Uncertainty and calibration of PORT with 3-seed ensembling (*n* = 37,948 test encounters). (a) Mean predicted risk $\left(\overset{‾}{p}\right)$ versus cross-seed standard deviation (*σ*_*p*_) by IoD label. (b) Calibration on log–log axes using log-spaced predicted-risk bins: observed IoD+ rate versus mean predicted risk (95% Wilson CIs). Predictions closely follow the diagonal, with slight upward deviation in the lowest-risk bins due to the irreducible baseline event rate.

**Figure 4:**
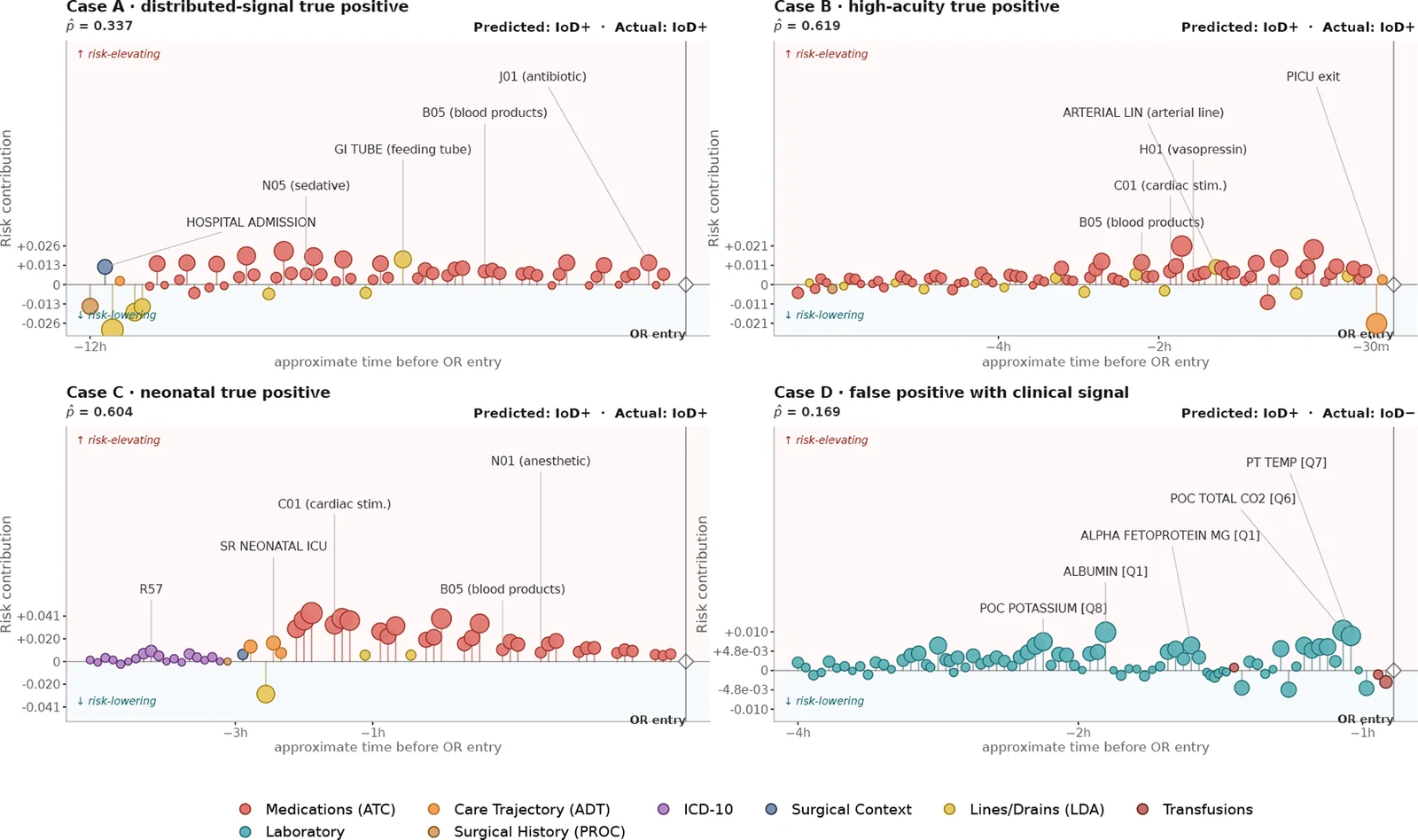
Per-encounter Integrated Gradients attribution for four illustrative test encounters. Signed per-token IG is shown over the 80 most recent clinical sequence positions (excluding administrative registration and ATC/ICD hierarchy-fragment tokens), with approximate time before OR entry on the *x*-axis and the white diamond marking OR entry. Marker size scales with |IG|, color indicates token category, and signed IG separates risk-elevating (red) from risk-lowering (blue) contributions. Up to five positive-IG tokens are annotated with brief clinical hints; all positive and negative attributions are displayed.

**Figure 5:**
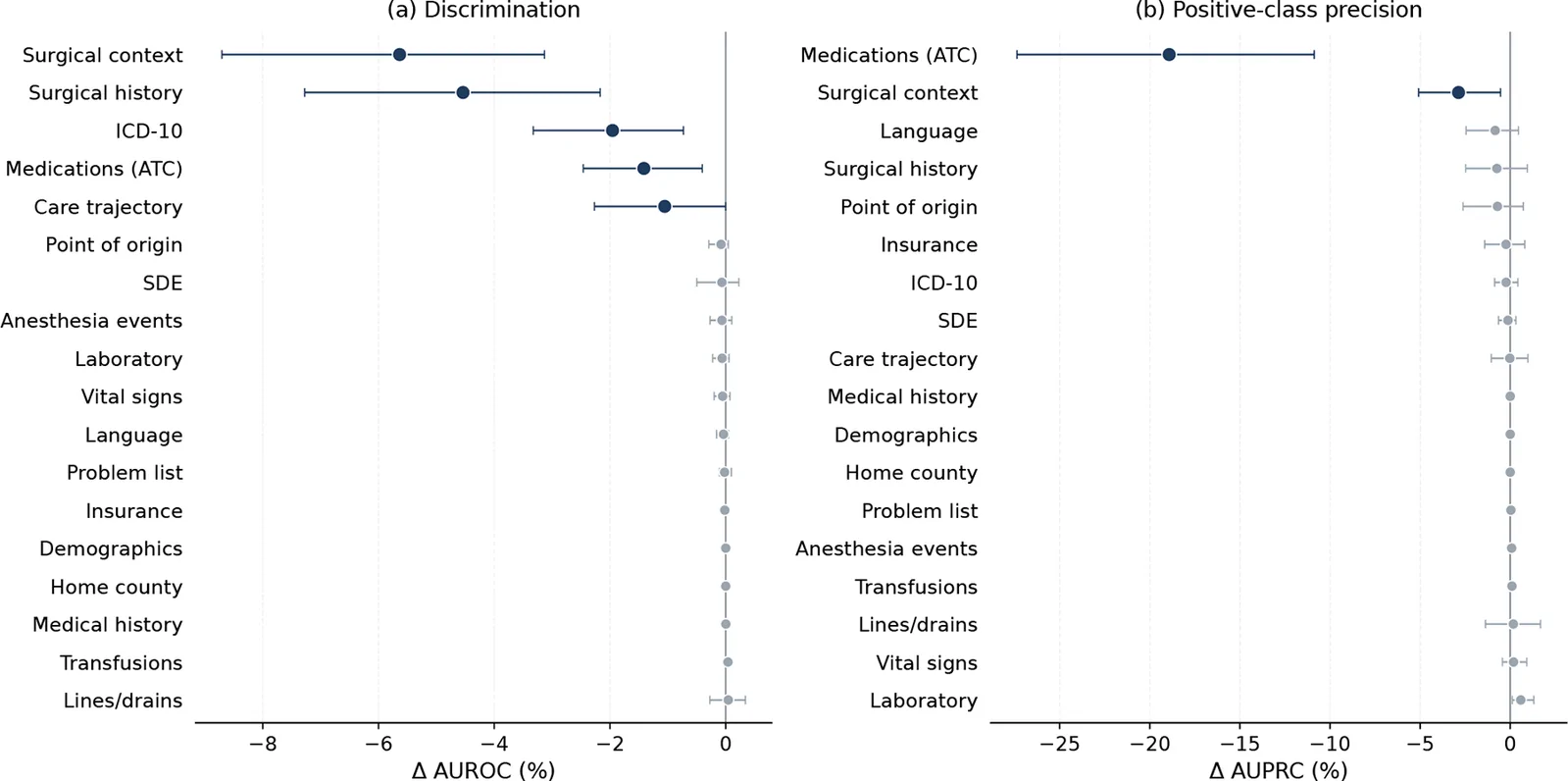
Category-level occlusion analysis. Forest plots show the change in (a) AUROC and (b) AUPRC after masking each token category and its adjacent quantile tokens (95% bootstrap CIs, 2,000 resamples; baseline AUROC 0.942, AUPRC 0.237). Categories are ranked independently by metric; those with a statistically significant, materially large effect are shown in navy, with the rest greyed. Medications (ATC) have the largest impact on AUPRC despite only a modest effect on AUROC. Per-category values are listed in the main text and Supplementary Table S13.

**Figure 6:**
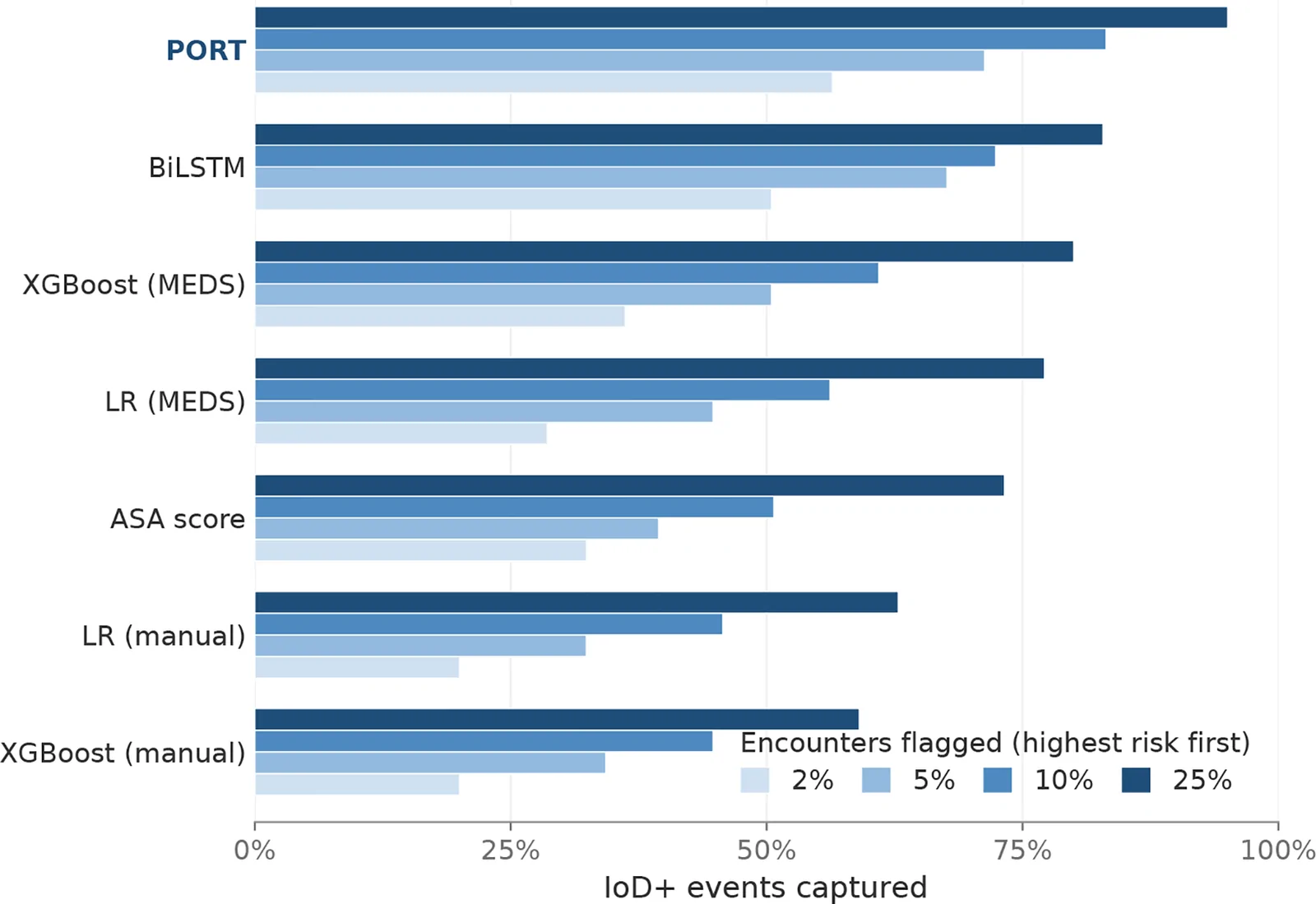
Event-capture by flagging fraction on the held-out test set. For each model, the four bars give the proportion of all IoD+ events captured when its highest-risk 2%, 5%, 10%, and 25% of encounters are flagged (light to dark). PORT captures the most IoD events at every flagging fraction (e.g., 83% within its highest-risk 10%, versus 72% for the next-best BiLSTM and ≤61% for the tabular and clinical models). Cohorts: PORT *n*=37,948 (101 IoD+); MEDS/manual baselines *n*=42,008 (105 IoD+); ASA *n*=23,957 (71 IoD+).

**Table 1:** Held-out test-set IoD prediction. Models are evaluated on the same held-out set of 37,948 backbone-scoreable encounters (101 IoD+, 0.27%); 4,060 unscoreable encounters are excluded. The ASA-only baseline is restricted to 23,957 encounters with a pre-operative ASA score (71 IoD+). AUROC and AUPRC are reported with patient-clustered bootstrap 95% confidence intervals (2,000 resamples). Layers indicate clinical variables (1), manual features (2), MEDS representations (3), and backbone readouts (4). LR, logistic regression; XGB, XGBoost; BiLSTM, bidirectional LSTM.

Layer	Model	AUROC ↑	AUPRC ↑	Brier ↓	ECE ↓	BSS ↑
1	ASA score	0.816 [.775, .857]	0.022 [.012, .045]	0.0029	0.0005	0.015
2	LR (manual)	0.775 [.722, .823]	0.025 [.013, .049]	0.0026	0.0006	0.009
2	XGBoost (manual)	0.757 [.703, .810]	0.021 [.011, .044]	0.0026	0.0007	0.006
3	LR (MEDS)	0.829 [.777, .877]	0.033 [.019, .057]	0.0026	0.0008	0.003
3	XGBoost (MEDS)	0.882 [.845, .916]	0.097 [.055, .169]	0.0026	0.0005	0.013
3	BiLSTM (MEDS sequences)	0.906 [.874, .934]	0.191 [.119, .282]	0.0024	0.0014	0.104
4	Backbone, zero-shot	0.588 [.522, .655]	0.057 [.020, .113]	0.0976	0.298	-
4	Backbone + linear probe	0.800 [.752, .844]	0.089 [.043, .155]	0.0031	0.0045	-
4	**PORT (Backbone + LoRA)**	**0.942 [.915, .964]**	**0.237 [.156, .322]**	**0.0023**	0.0014	**0.116**

**Table 2:** PORT operating characteristics across clinically motivated thresholds on the held-out test set (*n*=37,948, 101 IoD+). Thresholds are selected to achieve fixed sensitivity (≥70/80/85/90%), fixed specificity (≥80/90/95%), or Youden’s *J* (population-level optimum). *N* flagged is the number of encounters at or above the threshold.

Operating point	Threshold	Sens. ↑	Spec. ↑	PPV ↑	NPV ↑	*N* flagged
Sens. ≥ 90%	0.0004	0.901	0.886	0.021	1.000	4,392
Sens. ≥ 85%	0.0006	0.851	0.898	0.022	1.000	3,932
Sens. ≥ 80%	0.0010	0.802	0.919	0.026	0.999	3,132
Sens. ≥ 70%	0.0051	0.703	0.964	0.049	0.999	1,445
Youden’s *J*	0.0003	0.921	0.871	0.019	1.000	4,986
Spec. ≥ 80%	0.0002	0.941	0.841	0.016	1.000	6,098
Spec. ≥ 90%	0.0007	0.832	0.908	0.024	1.000	3,558
Spec. ≥ 95%	0.0040	0.713	0.958	0.043	0.999	1,667

**Table 3:** Patient and encounter characteristics across the analysis cohort (127,874 patients, 189,704 encounters), stratified by intraoperative-deterioration (IoD) outcome. Continuous variables shown as median (IQR); categorical variables as *n* (%). *p*-values from Mann–Whitney *U* for continuous variables and chi-square test for categorical variables. Gestational age available in 83.5% of IoD+ and 60.3% of IoD− encounters; percentages within that subset.

Characteristic	IoD− (*n*=189,255)	IoD+ (*n*=449)	*p*
Age at OR entry, years	6.6 (2.3–12.3)	2.4 (0.3–10.1)	<0.001
Weight, kg	22.1 (12.5–44.9)	11.8 (4.2–34.4)	<0.001
Height, cm	117.1 (86.4–150.1)	81.0 (52.1–130.0)	<0.001
Gestational age at birth, weeks	39.0 (36.0–40.0)	38.0 (35.0–40.0)	0.005
**Sex**			
Male	110,235 (58.2%)	252 (56.1%)	0.388
Female	79,017 (41.8%)	197 (43.9%)	
**Race**			
White	100,234 (53.0%)	207 (46.1%)	<0.001
Black or African American	60,046 (31.7%)	183 (40.8%)	
Asian	6,812 (3.6%)	18 (4.0%)	
Other / multiple	11,206 (5.9%)	27 (6.0%)	
Declined / unknown	10,957 (5.8%)	14 (3.1%)	
**Ethnicity**			
Hispanic or Latino	26,843 (14.2%)	48 (10.7%)	0.052
Non-Hispanic or Latino	161,865 (85.5%)	401 (89.3%)	
Declined / unknown	544 (0.3%)	0 (0.0%)	
**ASA Physical Status**			
I	46,060 (24.3%)	4 (0.9%)	<0.001
II	78,907 (41.7%)	46 (10.2%)	
III	56,453 (29.8%)	198 (44.1%)	
IV	4,879 (2.6%)	159 (35.4%)	
V	133 (0.1%)	21 (4.7%)	
VI	76 (0.0%)	18 (4.0%)	
**Patient class**			
Inpatient	49,227 (26.0%)	410 (91.3%)	<0.001
Ambulatory surgery	93,981 (49.7%)	11 (2.4%)	
Observation	41,523 (21.9%)	12 (2.7%)	
Outpatient	3,813 (2.0%)	5 (1.1%)	
Other	711 (0.4%)	11 (2.4%)	
**Procedure urgency**			
Elective	161,596 (85.4%)	367 (81.7%)	<0.001
Urgent (>60 min)	24,505 (12.9%)	58 (12.9%)	
Emergent (<60 min)	2,515 (1.3%)	23 (5.1%)	
Unknown / other	639 (0.3%)	1 (0.2%)	

**Table 4:** IoD composite label components with detailed operational definitions and the originating clinical data source (Supplementary Table S5).

Comp.	Definition	Data source	N pos.	Rate
IoD1	Cardiac arrest requiring CPR	Anesthesia events (CPR)	74	0.04%
IoD2	Quick Note keyword match (31 terms, LLM-screened, clinician-adjudicated)	Anesthesia events (Quick Note)	91	0.05%
IoD3	IV vasoactive bolus: epinephrine, phenylephrine (>2 doses), ephedrine (>2 doses), vasopressin, adenosine, atropine (age >3 mo)	Medication administration	124	0.07%
IoD4	Vasoactive infusion started or rate increased >100%: dopamine, epinephrine, milrinone, norepinephrine, phenylephrine, vasopressin	Medication administration	138	0.07%
IoD5	New arterial line placed after surgical incision (after procedure start time)	Lines/drains/airways (LDA)	92	0.05%
**IoD**	**Composite (any of IoD1–IoD5)**		**449**	**0.24%**

**Table 5:** IoD2 keyword filtering cascade. “Notes” = individual Quick Notes matching keywords; “IoD2 (enc.)” = unique encounters with ≥1 kept note (encounter-level label). Multiple notes may correspond to the same anesthesia encounter; values in parentheses denote notes removed at that step. Step 2 is a majority-vote screen of the Step-1-surviving candidate encounters by a three-model LLM ensemble under a rescue-versus-managed rubric; Steps 3–4 apply a few-shot re-screen and independent adjudication by two pediatric anesthesiologists.

Step	Notes	IoD2 (enc.)	Key exclusions
Keyword match (broad)	6,535	3,502	–
Step 1: Regex exclusion	3,805	1,785	“epi” → epidural (2,344 notes)
	(−2,730)		ophthalmic phenylephrine (94)
			“echo” → scheduling notes (67)
			planned hypotension (38)
Step 2: 3-model LLM ensemble	–	284	rescue-vs-managed rubric on Quick Note text, majority ≥2 of 3: excludes topical/local epinephrine & phenylephrine, transport/handoff, echo & imaging logistics, ECMO-flow maintenance, planned circulatory arrest (DHCA) and protocolized cardiac events, self-resolved/benign-rhythm, positioning artifact
Step 3: Few-shot re-screen	–	159	adds operation context + 18 worked examples; flagged for expert review
Step 4: Clinician adjudication	–	91	both-reviewer 59 + 28 disagreements + 4 cardiac
Final IoD2 (analysis cohort)	–	91	composite IoD = 449 analysis-cohort encounters

## Data Availability

Source EHR data are not publicly available because they contain protected health information. De-identified tokenized timelines and trained model weights can be shared on request to the corresponding author subject to a data use agreement with Children’s Healthcare of Atlanta. All analysis code, including the MEDS conversion pipeline, tokenization configuration, model training and inference scripts, baselines, and evaluation routines, is available at https://github.com/Kamaleswaran-Lab/PORT.
