# Supplementary material for: PORT: Generative EHR Pretraining for Pediatric Intraoperative Risk Prediction
Source: Res Sq. 2026 Aug 12:rs.3.rs-10609619. Preprint. [Version 1] doi: 10.21203/rs.3.rs-10609619/v1 (PMC13640902; doi:10.21203/rs.3.rs-10609619/v1)
Supplement: Supplementary file 1 [file NIHPPRS10609619V1-supplement-1.pdf]

# Supplementary Materials

## S1 Congenital Cardiac Surgery Definition and Procedure Classification

Congenital heart disease is among the strongest determinants of pediatric perioperative deterioration, and congenital cardiac operations form a uniformly high-acuity surgical population. A natural concern is therefore whether PORT's discrimination reflects genuine risk stratification or merely the identification of cardiac-surgery patients. To address this, we evaluated performance separately in cardiac and noncardiac surgery (Supplementary Table S2), which required an explicit and clinically defensible definition of congenital cardiac surgery.

We defined cardiac surgery by the operating surgeon rather than by procedure-name keywords. In the anesthesia information management system we restricted attention to the surgical service responsible for congenital cardiac surgery (cardiothoracic surgery) and identified its attending surgeons; eight surgeons accounted for essentially all congenital cardiac operations at the institution, each with a caseload that was 84–98% congenital cardiac procedures and a sharp discontinuity to the next-highest surgeon (below 1%). A board-certified pediatric anesthesiologist on the study team reviewed and confirmed this eight-surgeon roster. An encounter was labeled congenital cardiac surgery when its primary surgeon belonged to the roster, yielding 4,254 of 189,704 encounters in the analysis cohort and 854 of 37,948 in the held-out test set. This rule is fully reproducible and avoids the ambiguity of mapping free-text procedure names.

To make the definition transparent and verifiable, we enumerated every unique operation recorded for the cardiothoracic service (117 distinct procedure names) and independently labeled each against the STS-EACTS Congenital Heart Surgery (STAT) nomenclature[55] and the RACHS-1 categories,[8] by anatomic target and surgical intent rather than service line.[56] Operations on the heart or intrathoracic great vessels, including surgical rhythm-device and mechanical circulatory support procedures,[57] were labeled congenital cardiac surgery (58 procedures), whereas general-thoracic, transcatheter, electrophysiology, and staged-access procedures were excluded (59 procedures; Supplementary Table S1). This anatomic classification agreed with the surgeon-based definition on 99.8% of encounters (Cohen's $\kappa$=0.96); the small residual discordance was bidirectional and clinically interpretable, reflecting general-thoracic operations performed by the cardiac surgeons (e.g., pectus repair, diaphragm plication; $n$=176) and patent ductus arteriosus ligations performed by general pediatric surgeons outside the roster ($n$=158).

Evaluated under this definition, PORT's discrimination was preserved in the much larger noncardiac stratum (AUROC 0.932) while remaining weaker across all models within the uniformly high-acuity cardiac-surgery stratum (PORT 0.773); these findings were essentially unchanged when the anatomic procedure-name definition was substituted (Supplementary Table S2), indicating that PORT's headline performance is not an artifact of identifying cardiac-surgery patients.

Table S1: Classification of the 117 unique cardiothoracic-service procedure names into congenital cardiac surgery (58) versus noncardiac (59), used as the anatomic cross-check of the surgeon-based definition (Supplementary Section S1). Procedure names are lightly normalized from the scheduling system for readability.

| **Cardiac surgery (n = 58)** | **Excluded as noncardiac (n = 59)** |
|---|---|
| Repair ventricular septal defect (VSD) | Repair pectus excavatum |
| Repair atrial septal defect (ASD) | Repair pectus carinatum |
| Repair tetralogy of Fallot, complete (TOF) | Thoracoscopy pectus excavatum (Nuss) |

| Cardiac surgery (n = 58) | Excluded as noncardiac (n = 59) |
|---|---|
| Bidirectional Glenn anastomosis | Plication diaphragm |
| Repair coarctation of aorta | Thoracotomy |
| Repair complete atrioventricular canal | Explore chest |
| Fontan | Exploratory laparotomy |
| Pulmonary valve replacement (PVR) | Resection of teratoma |
| Blalock–Taussig shunt (BT) | Sternotomy |
| Arterial switch | Aortopexy |
| Resection of subaortic membrane | Excision chest mass/lesion, minor |
| Norwood | Secondary wound closure |
| Repair total or partial anomalous venous return | Flap, myocutaneous (any) |
| Repair vascular ring | Remove sternal wire |
| RV–PA conduit replacement | Closure chest |
| Insertion pacing system (any) | Aborted case |
| Mitral valve replacement/repair (MVR) | Insertion central venous catheter |
| Right ventricle to pulmonary artery conduit | Removal CVL |
| Pulmonary artery banding | Insertion gastrostomy tube |
| Repair partial atrioventricular canal | Gastrostomy, laparoscopic |
| Heart transplant | Placement G-tube, laparoscopic |
| Ligation patent ductus arteriosus (PDA) | Nissen fundoplication |
| Repair double-outlet right ventricle | Nissen fundoplication, laparoscopic |
| Repair right ventricular outflow tract obstruction | Nissen fundoplication, laparoscopic with G-tube |
| Replacement pacemaker generator | Pyloromyotomy, laparoscopic |
| Pulmonary artery plasty | Ladd procedure |
| Anomalous left coronary artery from pulmonary artery | Ladd, laparoscopic |
| Mitral valve repair | Colectomy, laparoscopic |
| Ross | Colostomy |
| Aortic valve replacement (AVR) | Revision/closure colostomy |
| Rastelli | Repair duodenal atresia |
| Aortic arch augmentation vs. repair | Anoplasty |
| Sinus venosus ASD | Anorectoplasty |
| Insertion ventricular assist device | Herniorrhaphy, laparoscopic inguinal |
| Repair truncus arteriosus | Laparoscopy, diagnostic |
| Repair aortic arch | Biopsy liver, percutaneous |
| Tricuspid valve repair vs. TVR | EGD |
| Aortic valve replacement vs. repair | Biopsy with EGD |
| Interrupted aortic arch repair | Laryngoscopy/bronchoscopy, direct |
| ECMO decannulation | Diagnostic laryngoscopy/bronchoscopy/supraglottoplasty |
| Reimplantation coronary artery | Laryngoscopy, micro |
| Mitral valve replacement | Bronchoscopy, flexible |
| Fluoroscopy-guided pulmonary artery plasty | Laryngotracheoplasty |
| ECMO cannulation | Tracheostomy |
| Biventricular pacer | Tonsillectomy/adenoidectomy |
| Aortic valve repair | Adenoidectomy |
| Repair transposition of great arteries (TGA) | Nephrectomy |
| Repair aorta | Cystoscopy |
| Aortic arch replacement | Repair testicular torsion |
| Septal myomectomy | Endoscopic third ventriculostomy |
| Double switch repair | Lensectomy/anterior vitrectomy/IOL placement |
| Damesk–Stancil–Kaye (DSK) | Restoration, dental |
| Mee shunt | Closed reduction/percutaneous pinning, clavicle |
| A–P window | Aspiration, bone marrow |
| Insertion cardiac defibrillator (AICD) | Biopsy mass |
| Konno | Exploration femoral artery |
| Reimplantation of subclavian | Transesophageal echo (TEE) |
| Pericardial window | MRI |
| | IRAD (unresolvable; default-excluded) |

Table S2: Surgery-type–stratified test-set IoD performance, with held-out predictions re-evaluated within each stratum without retraining. Cardiac surgery is defined by the operating surgeon (Supplementary Section S1); all other encounters form the noncardiac stratum. Stratum sizes (cardiac / noncardiac): PORT-class readouts ($n$=37,948), 854 (31 IoD+) / 37,094 (70 IoD+); MEDS and manual baselines ($n$=42,008), 854 / 41,154; ASA ($n$=23,957), 785 / 23,172. AUROC and AUPRC with patient-clustered bootstrap 95% CIs (1,000 resamples). The cardiac-stratum AUPRC is inflated by the ∼3.6% event rate rather than improved ranking.

| | Noncardiac surgery | | Cardiac surgery | |
|---|---|---|---|---|
| **Model** | **AUROC ↑** | **AUPRC ↑** | **AUROC ↑** | **AUPRC ↑** |
| ASA score | 0.785 [.735, .835] | 0.011 [.005, .027] | 0.664 [.560, .755] | 0.066 [.028, .163] |
| LR (manual) | 0.730 [.665, .789] | 0.017 [.007, .048] | 0.546 [.432, .645] | 0.065 [.031, .120] |
| XGBoost (manual) | 0.708 [.644, .773] | 0.015 [.006, .044] | 0.557 [.443, .680] | 0.054 [.030, .113] |
| LR (MEDS) | 0.775 [.708, .836] | 0.020 [.010, .042] | 0.696 [.610, .779] | 0.086 [.046, .183] |
| XGBoost (MEDS) | 0.847 [.798, .890] | 0.090 [.041, .177] | 0.698 [.596, .789] | 0.128 [.062, .267] |
| BiLSTM (MEDS sequences) | 0.871 [.828, .908] | 0.190 [.108, .290] | 0.784 [.698, .862] | 0.193 [.097, .326] |
| Backbone, zero-shot | 0.570 [.492, .650] | 0.051 [.009, .114] | 0.623 [.492, .730] | 0.138 [.055, .283] |
| Backbone + linear probe | 0.793 [.734, .848] | 0.088 [.029, .165] | 0.715 [.614, .807] | 0.141 [.074, .285] |
| **PORT** | **0.932 [.895, .962]** | **0.248 [.143, .361]** | **0.773 [.685, .848]** | **0.224 [.116, .389]** |

## S2 LoRA Hyperparameter Sweep

To select the LoRA configuration for the main results, we performed a $5 \times 4$ grid over LoRA rank ($r \in \{4, 8, 16, 32, 64\}$, with $\alpha = 2r$) and four loss formulations: (i) weighted BCE with $w_+ = n_-/n_+ \approx 446$ (class-balanced default for rare-event tasks); (ii) unweighted BCE ($w_+ = 1$); (iii) focal loss with $\gamma$=2, $\alpha$=0.25[58]; and (iv) positive-class oversampling at 5× combined with weighted BCE. Each of the 20 cells was fine-tuned on the pretrained backbone for 15 epochs with patience 5 (val AUROC), single seed 123, otherwise identical hyperparameters to the main PORT run. Supplementary Table S3 reports test-set AUROC, AUPRC, and Brier point estimates for all 20 cells plus two focal-$\alpha$=0.75 verification cells.

Across the grid, AUPRC is concentrated in a narrow band: the five highest-AUPRC cells of the $5 \times 4$ grid, ($r$=16, BCE), ($r$=8, BCE), ($r$=4, BCE), ($r$=4, oversample), and ($r$=8, focal), span only 0.231–0.242 in point estimate, indicating that performance is robust to LoRA capacity and loss choice within reasonable ranges. We selected ($r$=8, unweighted BCE) as the most balanced configuration rather than the leader on any single metric: no cell was simultaneously best on all three metrics, whose maxima fell in three different configurations—AUROC 0.946 at ($r$=8, focal), AUPRC 0.242 at ($r$=16, BCE), and Brier 0.0023 at ($r$=32, BCE). The selected cell lay within 0.01 of each of these maxima (AUROC 0.936, AUPRC 0.241, Brier 0.0024), giving the strongest joint discrimination–calibration profile in the grid while requiring no class re-weighting. Two considerations favor unweighted BCE: (a) it avoids the auto-computed $w_+ \approx 446$, which is over-aggressive on the ≈0.3% prevalence task; and (b) focal $\alpha$=0.25 corresponds to the RetinaNet object-detection default and weights the rare positive class *less* than the abundant negative class, which is the opposite direction expected for IoD. We verified that re-running focal at $\alpha$=0.75 (positive-favoring) for $r \in \{4, 8\}$ worsened calibration (Brier 0.0075 and 0.0069 vs. 0.0024 for BCE) without improving discrimination (AUPRC 0.167 and 0.178). Positive-class oversampling at 5× degraded calibration across all ranks (Brier 0.0061–0.0081) and is reported only for completeness.

Table S3: LoRA hyperparameter sweep: the complete $5 \times 4$ grid plus two focal $\alpha$=0.75 verification cells, evaluated on the held-out test set under single seed 123. The selected PORT configuration is marked (✓) in the rightmost column; because no single cell was best on all three metrics simultaneously, selection favored the most balanced configuration (see text).

| **Rank** | **Loss** | **AUROC ↑** | **AUPRC ↑** | **Brier ↓** | **Selected** |
|---|---|---|---|---|---|
| $r$=4 | weighted BCE | 0.942 | 0.190 | 0.0027 | |
| $r$=4 | unweighted BCE | 0.934 | 0.236 | 0.0024 | |
| $r$=4 | focal $\alpha$=0.25 | 0.933 | 0.201 | 0.0026 | |
| $r$=4 | oversample 5× | 0.930 | 0.235 | 0.0076 | |
| $r$=8 | weighted BCE | 0.934 | 0.203 | 0.0026 | |
| $r$=8 | unweighted BCE | 0.936 | 0.241 | 0.0024 | ✓ |
| $r$=8 | focal $\alpha$=0.25 | 0.946 | 0.231 | 0.0024 | |
| $r$=8 | oversample 5× | 0.931 | 0.228 | 0.0061 | |
| $r$=16 | weighted BCE | 0.933 | 0.218 | 0.0025 | |
| $r$=16 | unweighted BCE | 0.927 | 0.242 | 0.0023 | |
| $r$=16 | focal $\alpha$=0.25 | 0.915 | 0.213 | 0.0034 | |
| $r$=16 | oversample 5× | 0.935 | 0.206 | 0.0071 | |
| $r$=32 | weighted BCE | 0.908 | 0.228 | 0.0023 | |
| $r$=32 | unweighted BCE | 0.929 | 0.225 | 0.0023 | |
| $r$=32 | focal $\alpha$=0.25 | 0.917 | 0.096 | 0.0027 | |
| $r$=32 | oversample 5× | 0.938 | 0.221 | 0.0081 | |
| $r$=64 | weighted BCE | 0.910 | 0.181 | 0.0027 | |
| $r$=64 | unweighted BCE | 0.918 | 0.174 | 0.0024 | |
| $r$=64 | focal $\alpha$=0.25 | 0.902 | 0.219 | 0.0052 | |
| $r$=64 | oversample 5× | 0.884 | 0.164 | 0.0063 | |
| Verification cells (focal $\alpha$=0.75, positive-favoring): | | | | | |
| $r$=4 | focal $\alpha$=0.75 | 0.939 | 0.167 | 0.0075 | |
| $r$=8 | focal $\alpha$=0.75 | 0.941 | 0.178 | 0.0069 | |

## S3 Data Efficiency: PORT vs BiLSTM under Decreasing Training Fraction

We assessed how PORT and the BiLSTM scale with training-set size by fitting both models at fractions $\{1, 5, 10, 25, 50, 100\}\%$ of the training cohort (stratified subsampling preserving the IoD+/IoD− ratio). PORT was fine-tuned at each fraction with the same LoRA configuration as the main result; the BiLSTM was trained at each fraction with the tuned configuration selected on full data ($h$=128, $l$=2, dropout 0.3) to maintain an apples-to-apples comparison. The held-out test set ($n$=37,948) was fixed across fractions for both models. Supplementary Fig. S1 plots AUROC and AUPRC against training fraction.

The two models scale differently. At intermediate fractions the tuned BiLSTM held a discriminative advantage (AUROC 0.874 vs PORT 0.793 at 25%; AUPRC 0.102 vs 0.045 at 25%), consistent with a supervised-from-scratch sequence architecture extracting more signal from a small labeled cohort than a large pretrained backbone whose LoRA adapter has too few positives to re-weight productively. The crossover occurred at 50% of training data, where PORT decisively exceeded BiLSTM on both metrics (AUROC 0.931 vs 0.883; AUPRC 0.142 vs 0.107) and continued to lead at full data (AUROC 0.942 vs 0.906, +0.036; AUPRC 0.237 vs 0.191, +0.046), with the largest gap on AUPRC where the pretrained representation pays off in identifying rare positives (main-text Table 1). The practical implication is that the generative-pretraining-plus-LoRA recipe reliably outperforms a from-scratch sequence model once the labeled cohort is large enough to drive the adapter (approximately half of our 132,671-encounter training pool); smaller institutions or rarer events may continue to benefit from a tuned supervised sequence baseline.

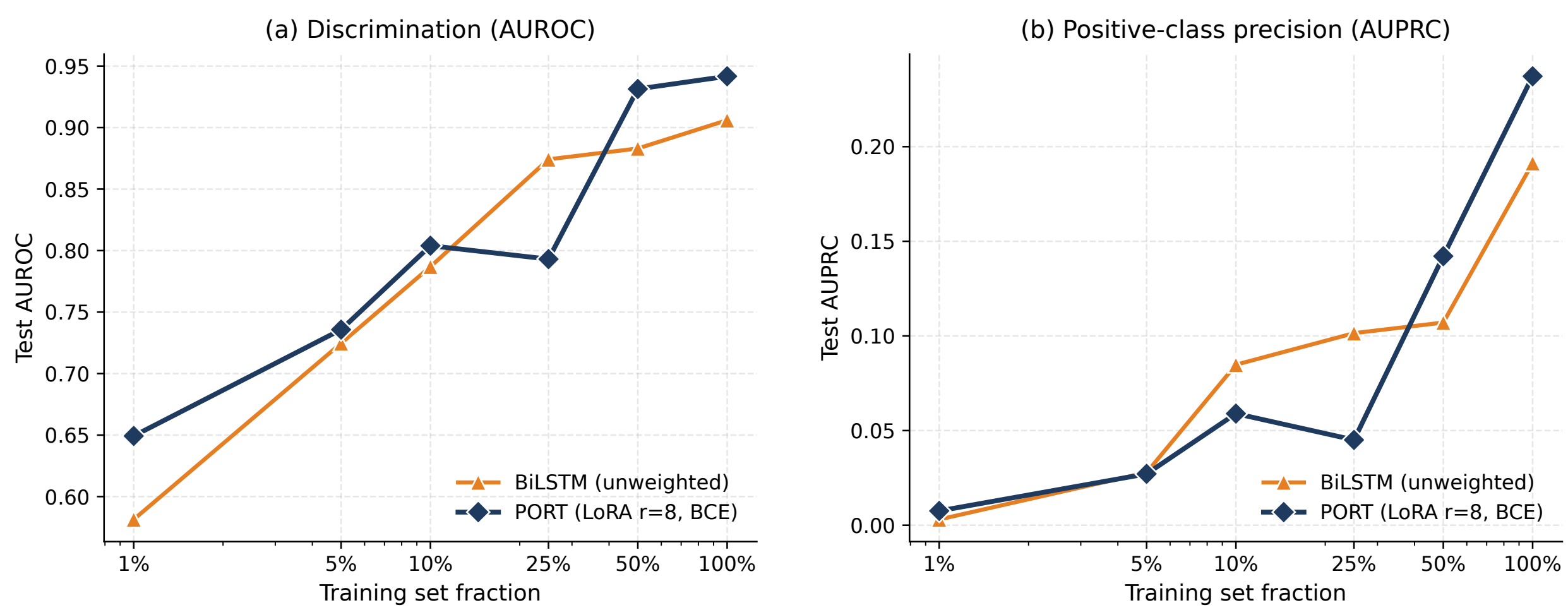


Figure S1: Data efficiency: PORT vs BiLSTM under decreasing training-set fraction ($\{1, 5, 10, 25, 50, 100\}\%$, log-spaced). (a) Test AUROC; (b) Test AUPRC. Both models trained with unweighted BCE; the BiLSTM uses its tuned configuration ($h$=128, $l$=2, dropout 0.3) at all fractions, with the held-out test set fixed across fractions.

# S4 Context-Window Ablation

To characterize how much pre-operative history PORT meaningfully leverages, we fixed the prediction point and 24 h generation horizon and varied only the look-back window across 7, 30, 90, and 365 days plus the patient's complete history. All models were re-trained and evaluated under each window with otherwise identical hyperparameters.

PORT achieved its highest AUPRC using full patient history (0.237), although a 7-day window already captured most of the available signal (AUPRC 0.212, AUROC 0.943; Supplementary Fig. S2). Across the 7-, 30-, 90-, and 365-day windows and full history, PORT AUPRC was 0.212, 0.148, 0.210, 0.184, and 0.237, and AUROC was 0.943, 0.916, 0.914, 0.893, and 0.942. AUROC was relatively insensitive to context length (PORT 0.893–0.943 across windows), and a 7-day window matched full-history AUROC (0.943 vs 0.942); PORT led all baselines on AUROC at every window. On AUPRC the tuned BiLSTM was comparable to PORT at short windows (0.217 vs 0.212 at 7 days) but fell below it at full history (0.191 vs 0.237), while tabular MEDS-aggregate models declined as the window lengthened. Practically, a 7-day window captures most of PORT's discriminative signal, with longer histories mainly improving rare-event (AUPRC) resolution. The deployment implication is that institutions without complete longitudinal histories can still expect near-peak discrimination from PORT given the most recent week of structured data.

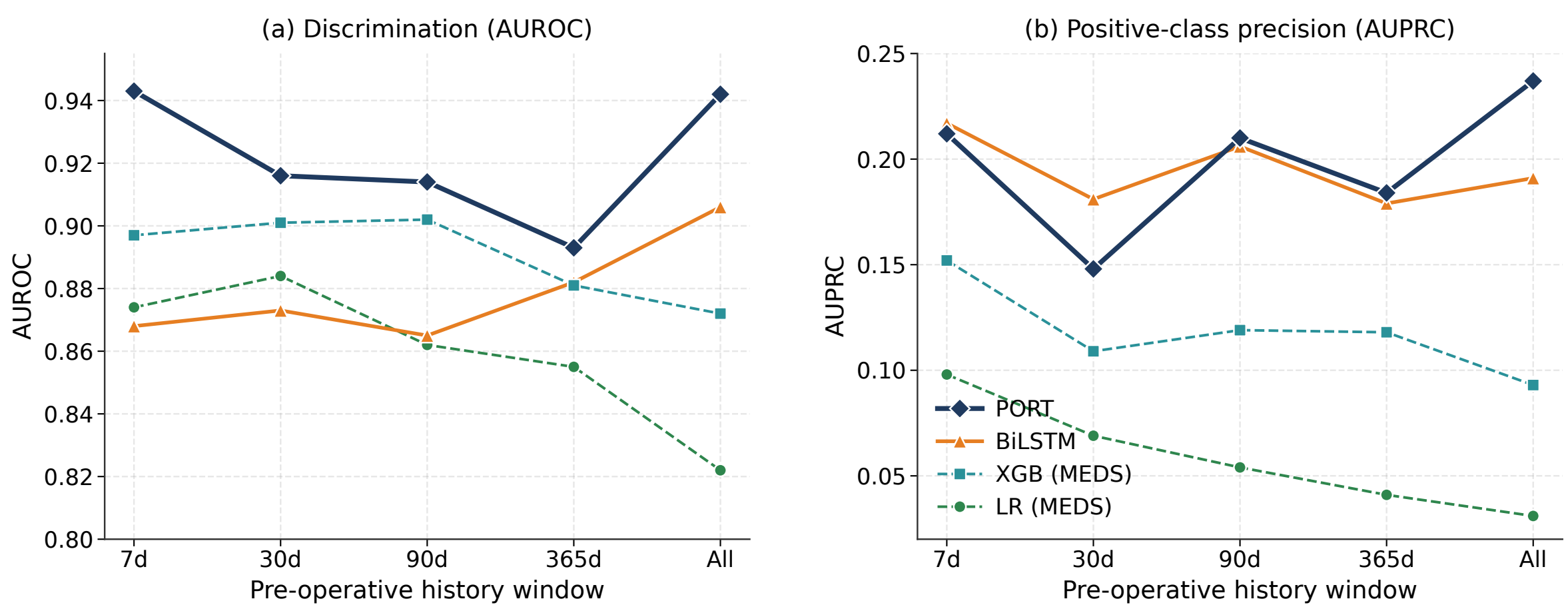


Figure S2: Context-window ablation. (a) AUROC and (b) AUPRC as a function of the pre-operative history available to each model. Bootstrap 95% CIs (2,000 resamples) are narrower than the between-window deltas of interest and omitted from the plot for clarity.

## S5 External-Time (Temporal) Validation

To assess temporal transportability under dataset shift, we re-partitioned the cohort by calendar time rather than at random. Patients first appearing before January 2019 formed the training set, whereas those first appearing in 2019 or later formed a temporally held-out test set containing entirely new patients. The generative backbone and all supervised models were retrained from scratch on the pre-2019 partition and evaluated on the post-2019 cohort using the common PORT-scoreable subset (53,992 encounters, 57 IoD+, prevalence 0.11%) with patient-clustered bootstrap 95% confidence intervals (2,000 resamples).

PORT retained excellent discrimination under temporal shift, achieving an AUROC of 0.949 (individual seeds 0.936–0.944), comparable to the internal test set (0.942) and higher than all baselines (BiLSTM, 0.877; XGBoost-MEDS, 0.847; Supplementary Table S4, Supplementary Fig. S3). In contrast, AUPRC decreased from 0.237 to 0.051. This reduction reflected the lower outcome prevalence in the temporal cohort (0.11% versus 0.27%), shorter pre-operative histories for newly encountered patients (median 102 versus 187 tokens; Supplementary Fig. S4), and a modest decline in precision among the highest-ranked predictions. Even after accounting for prevalence and context length, temporal AUPRC remained lower than that of the internal cohort, indicating a genuine loss of precision under temporal shift.

Despite this reduction, PORT remained the best-performing model in both discrimination and calibration. It achieved a Brier score of 0.0010, an expected calibration error of 0.0003, and was the only model to improve upon the prevalence baseline in terms of Brier Skill Score. Overall, these findings indicate that PORT generalizes well to future patient populations, although prospective deployment would require recalibration on contemporary data before high-confidence risk estimates are used operationally.

Table S4: External-time (temporal) validation. Models trained on pre-2019 patients and evaluated on temporally held-out post-2019 patients (entirely new patients; common PORT-scoreable subset 53,992 encounters, 57 IoD+, prevalence 0.11%). AUROC and AUPRC with patient-clustered bootstrap 95% CIs (2,000 resamples). PORT is reported as the three-seed probability ensemble (averaging the three fine-tuning seeds' predicted probabilities, as in the uncertainty analysis of Fig. 3); per-seed AUROC 0.936–0.944, AUPRC 0.028–0.049. Baselines are single deterministic fits. **Bold** = best. The low positive count makes AUPRC and BSS noisy; PORT is the only model whose Brier improves on the prevalence baseline (BSS $> 0$).

| Model | AUROC ↑ | AUPRC ↑ | Brier ↓ | ECE ↓ | BSS ↑ |
|---|---|---|---|---|---|
| LR (manual) | 0.788 [.718, .851] | 0.009 [.005, .019] | 0.0011 | 0.0018 | −0.028 |
| XGBoost (manual) | 0.780 [.709, .847] | 0.010 [.006, .024] | 0.0011 | 0.0016 | 0.000 |
| LR (MEDS) | 0.834 [.765, .894] | 0.010 [.006, .024] | 0.0012 | 0.0016 | −0.179 |
| XGBoost (MEDS) | 0.847 [.793, .897] | 0.009 [.005, .019] | 0.0011 | 0.0015 | 0.000 |
| BiLSTM (MEDS sequences) | 0.877 [.836, .913] | 0.020 [.009, .058] | 0.0045 | 0.0189 | −3.277 |
| Backbone + linear probe | 0.780 [.717, .834] | 0.010 [.003, .044] | 0.0011 | 0.0022 | −0.037 |
| **PORT (Backbone + LoRA)** | **0.949 [.919, .974]** | **0.051 [.027, .112]** | **0.0010** | **0.0003** | **0.025** |

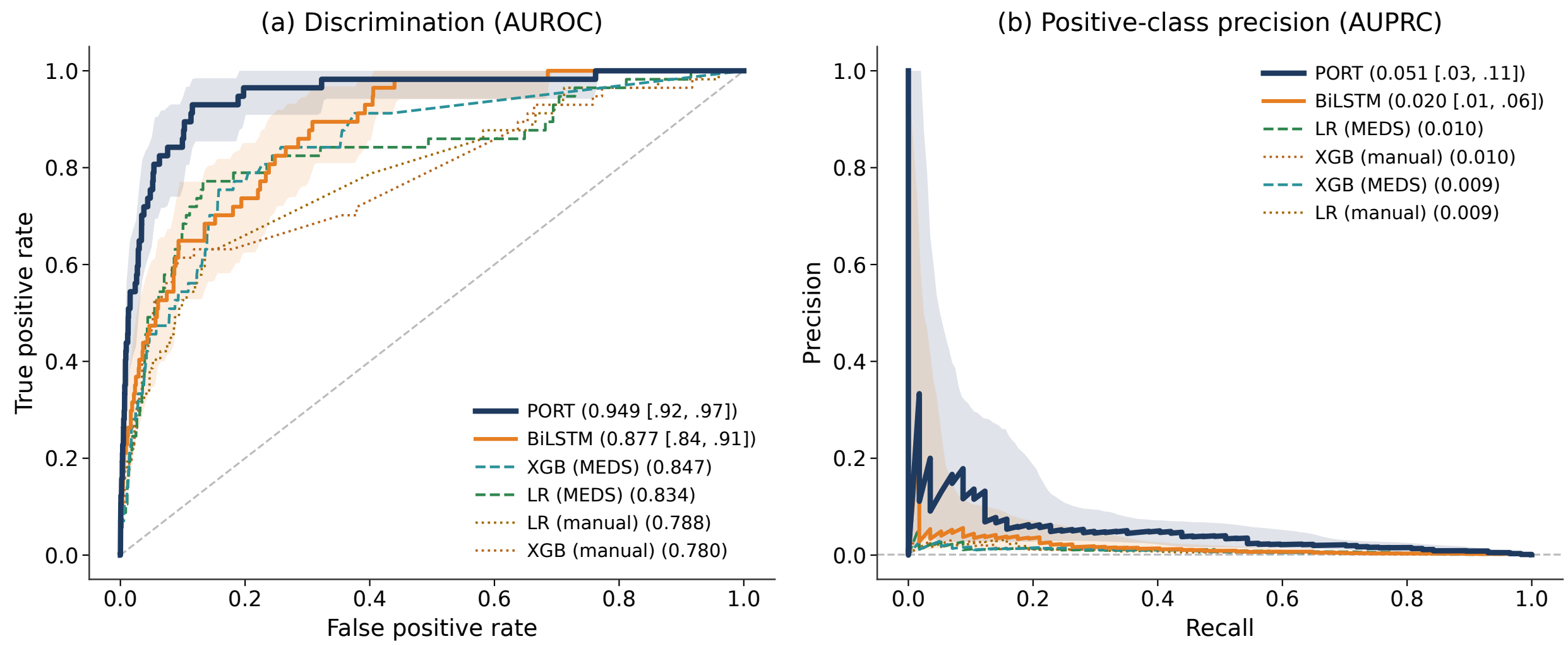


Figure S3: External-time (temporal) validation on post-2019 new patients (53,992 encounters, 57 IoD+). (a) ROC and (b) precision–recall curves on the common PORT-scoreable subset; legend values are AUROC/AUPRC with patient-clustered bootstrap 95% CIs (matching Supplementary Table S4). PORT is the three-seed probability ensemble (navy, bootstrap CI shaded); BiLSTM is shown with its bootstrap CI (orange). PORT's ranking transfers almost intact (AUROC 0.949 versus 0.942 internally) while absolute AUPRC is lower in this lower-prevalence, shorter-context future cohort (see text and Supplementary Fig. S4).

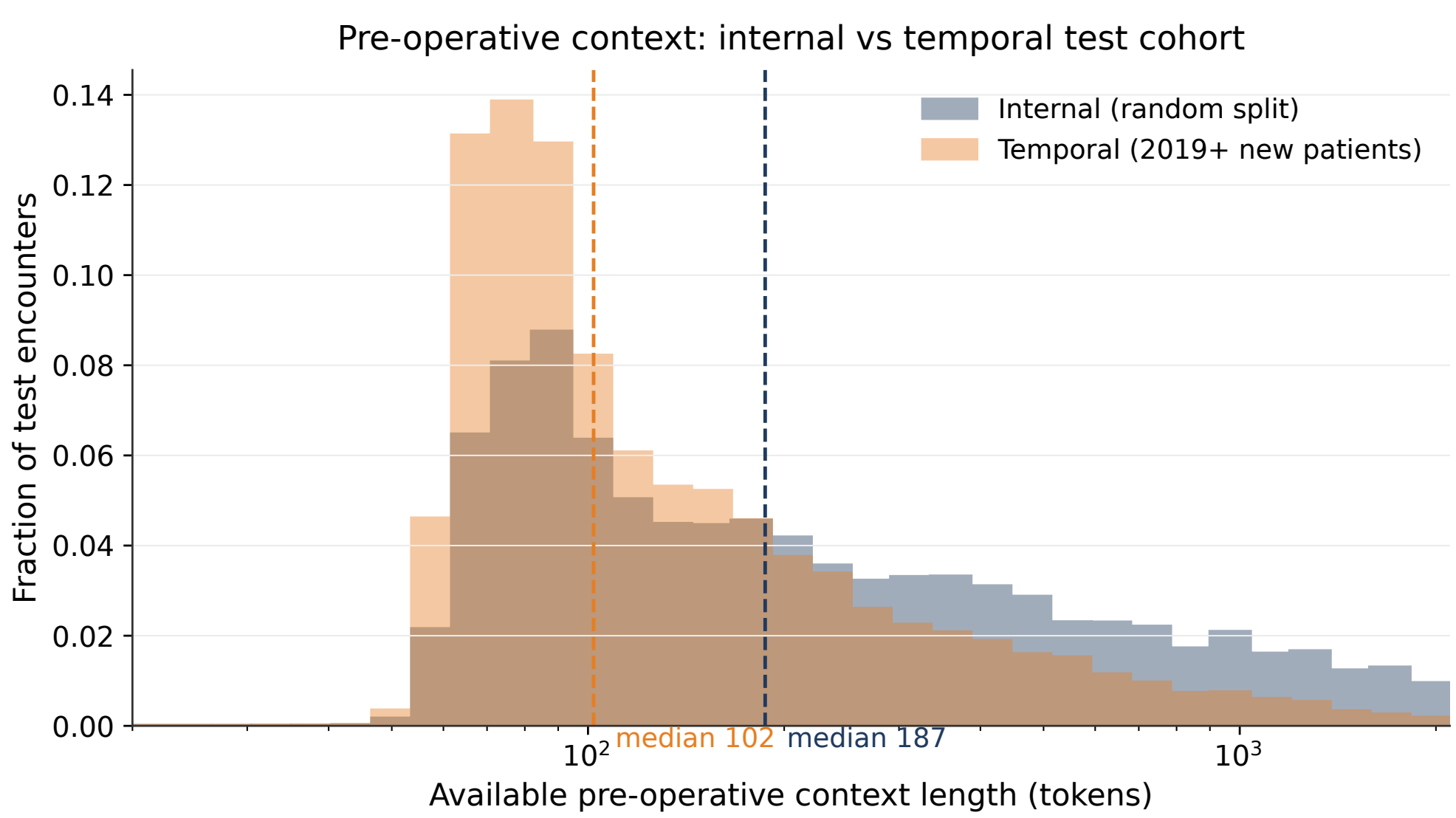


Figure S4: Distribution of available pre-operative context length (tokenized sequence length) in the internal random-split and temporal (post-2019 new-patient) test cohorts. The temporal cohort had substantially shorter pre-operative histories (median 102 versus 187 tokens; 82% versus 58% below 256 tokens), contributing to its lower AUPRC. Nevertheless, even among long-context encounters ($\geq$1024 tokens) with matched outcome prevalence (1.15% versus 1.13%), internal AUPRC (0.35) remained higher than temporal AUPRC (0.06), indicating an additional temporal distribution shift.

## S6 Extended Per-Encounter Attribution: 9 Cases Across TP / FP / TN

The four-case main-text presentation (Fig. 4) is illustrative rather than population-representative. To probe attribution behavior more broadly, we ran full-context IG on the entire held-out test cohort ($n$=37,948 encounters; ∼5 hours total on two H200 GPUs) and selected nine patients spanning three true positives, three false positives, and three confidently negative true negatives. The same patients are used in Supplementary Figures S5–S7 and Supplementary Fig. S8 so that long-context behavior and category-level aggregation can be read jointly.

**Long-context attribution distribution.** Supplementary Figures S5, S6, and S7 show the full-context per-token IG distribution as a dot plot for each of the nine patients; each clinical token is rendered as a dot at its true pre-OR position with color indicating token category, area indicating |IG|, and vertical position indicating the signed contribution to PORT's prediction. The white diamond at the right edge marks the current OR-entry position, with prior surgical encounters of the same patient (if any) shown as faded diamonds. In the cases with extended pre-operative records (spanning up to several years), attribution is distributed across the longitudinal timeline, but the largest contributions concentrate in the weeks to months immediately before OR entry, while more distant events each carry small, individually modest attributions. This pattern holds whether the eventual prediction is high (TP, FP) or low (TN). Acutely presenting cases (e.g., FP-2) instead carry only days of pre-operative history and are dominated entirely by recent events.

**Per-patient category aggregation.** Supplementary Fig. S8 aggregates per-token IG by category for the same nine patients. The bars sum the signed IG of every token within each vocabulary category, so that categories with bars to the right are net risk-elevating and bars to the left are net risk-lowering for that patient. Two patterns are clinically interpretable. (i) For the true positives (Cases TP-1–TP-3, each assigned high risk, $\hat{p}\approx$0.77–0.78), the largest positive category sums come from medications, laboratory values, ICD-10 diagnoses, and prior cardiac surgical history, consistent with high-acuity patients. (ii) For confident true negatives (Cases TN-1–TN-3), routine low-acuity tokens together with care-trajectory categories keep the prediction near the cohort floor ($\hat{p}\approx0$). False positives (Cases FP-1–FP-3, $\hat{p}$=0.58–0.69) show an elevated-risk category signature (medications, surgical history, and cardiac diagnoses) despite an encounter that proceeded without an IoD event, illustrating a limitation of attribution-based interpretability when high-risk markers are decoupled from the eventual intraoperative course.

**Honest caveats on token-level attribution.** Some risk-elevating tokens identified at the individual-patient level lack a clean clinical mechanism. For example, chronic-medication tokens such as a long-standing sedative regimen documented across the year before surgery can carry positive IG; this most plausibly reflects a chronic-illness marker (patients on long-term medications tend to be sicker) rather than a directly modifiable risk factor. Similarly, several "risk-lowering" tokens for true-negative patients (e.g., a peripheral IV removal) are routine procedural events that the model uses as low-acuity context rather than mechanistic interventions. We therefore present the per-encounter attribution as a tool for clinical interpretation and triage support, not as causal evidence; mechanism testing would require prospective intervention. Cohort-level category occlusion (Fig. 5) remains the appropriate evidence layer for cohort-wide importance.

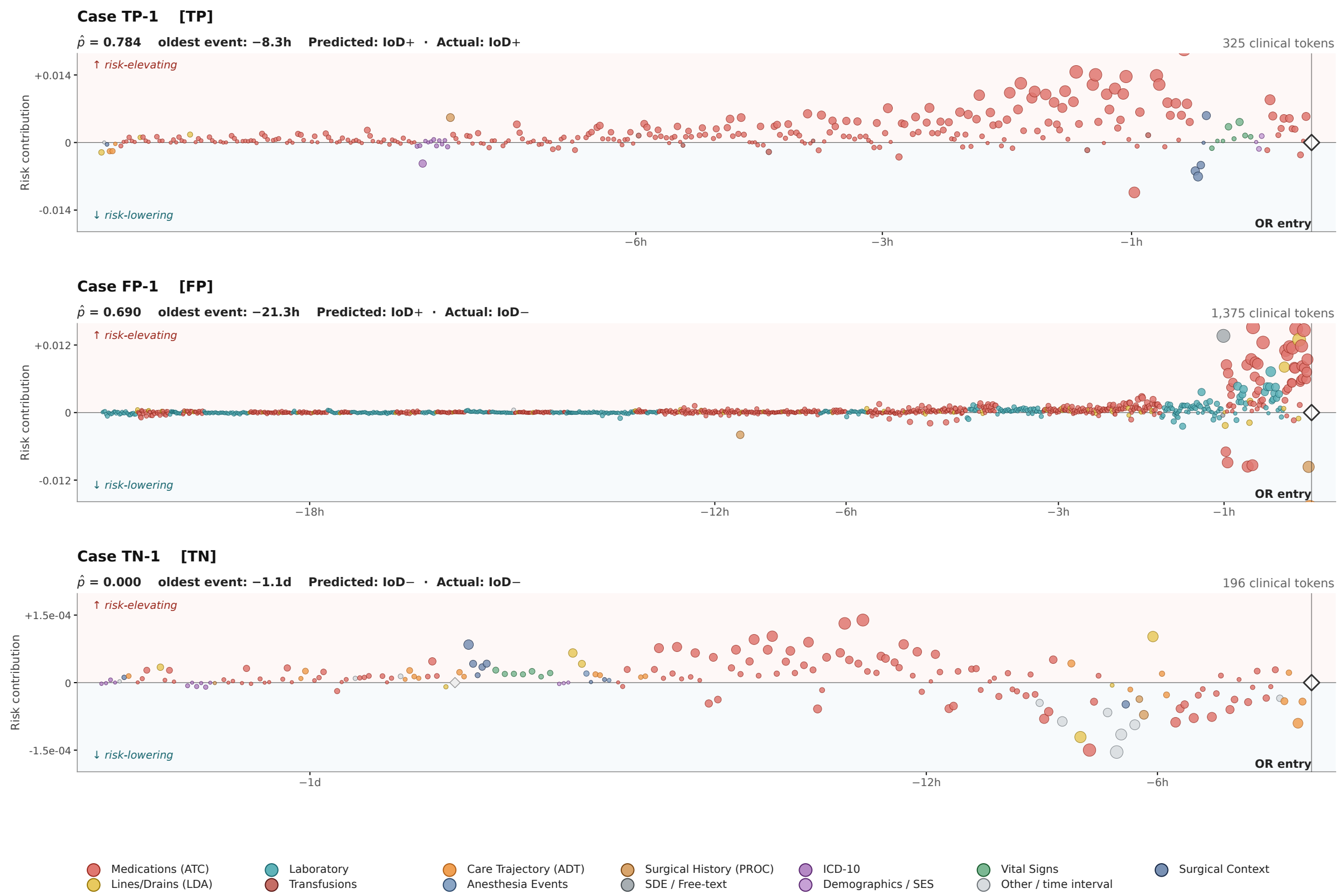


Figure S5: Extended per-encounter IG attribution, dot view (page 1 of 3): one true positive, one false positive, one true negative. Each panel shows every clinical token in the patient's full pre-OR timeline as a dot at its approximate time before OR entry; color encodes token category, area encodes |IG|, vertical position is the signed contribution. The white diamond at the right edge marks the current OR entry; faded diamonds (if present) mark OR entries of the patient's prior surgical encounters in the timeline. Header reports the case label (TP/FP/TN-{1–3}), the predicted-versus-actual outcome at the Youden's $J$ operating point (Table 2), the oldest event in the patient's per-encounter context, and the total number of clinical tokens.

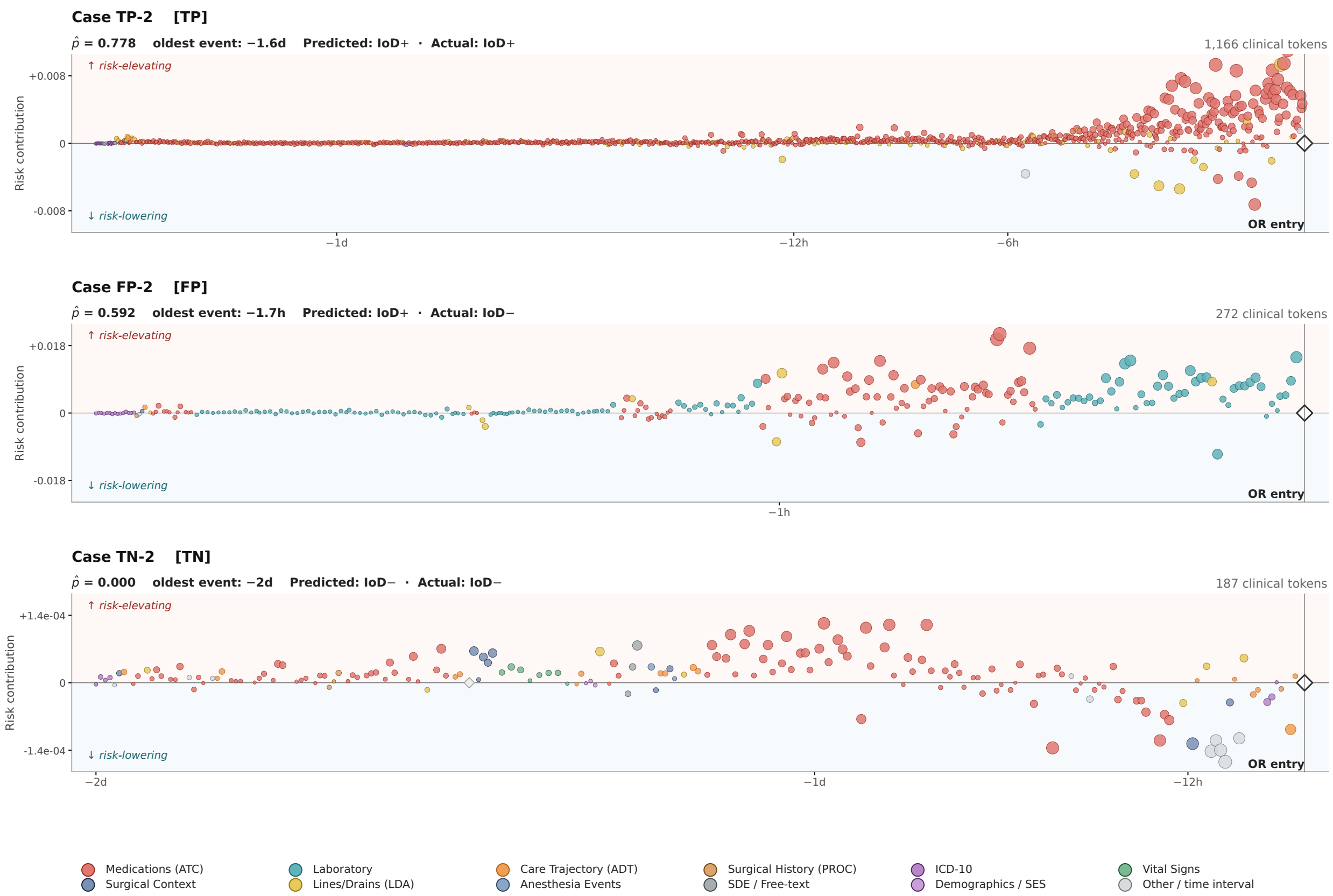


Figure S6: Extended per-encounter IG attribution, dot view (page 2 of 3). Layout as in Supplementary Fig. S5.

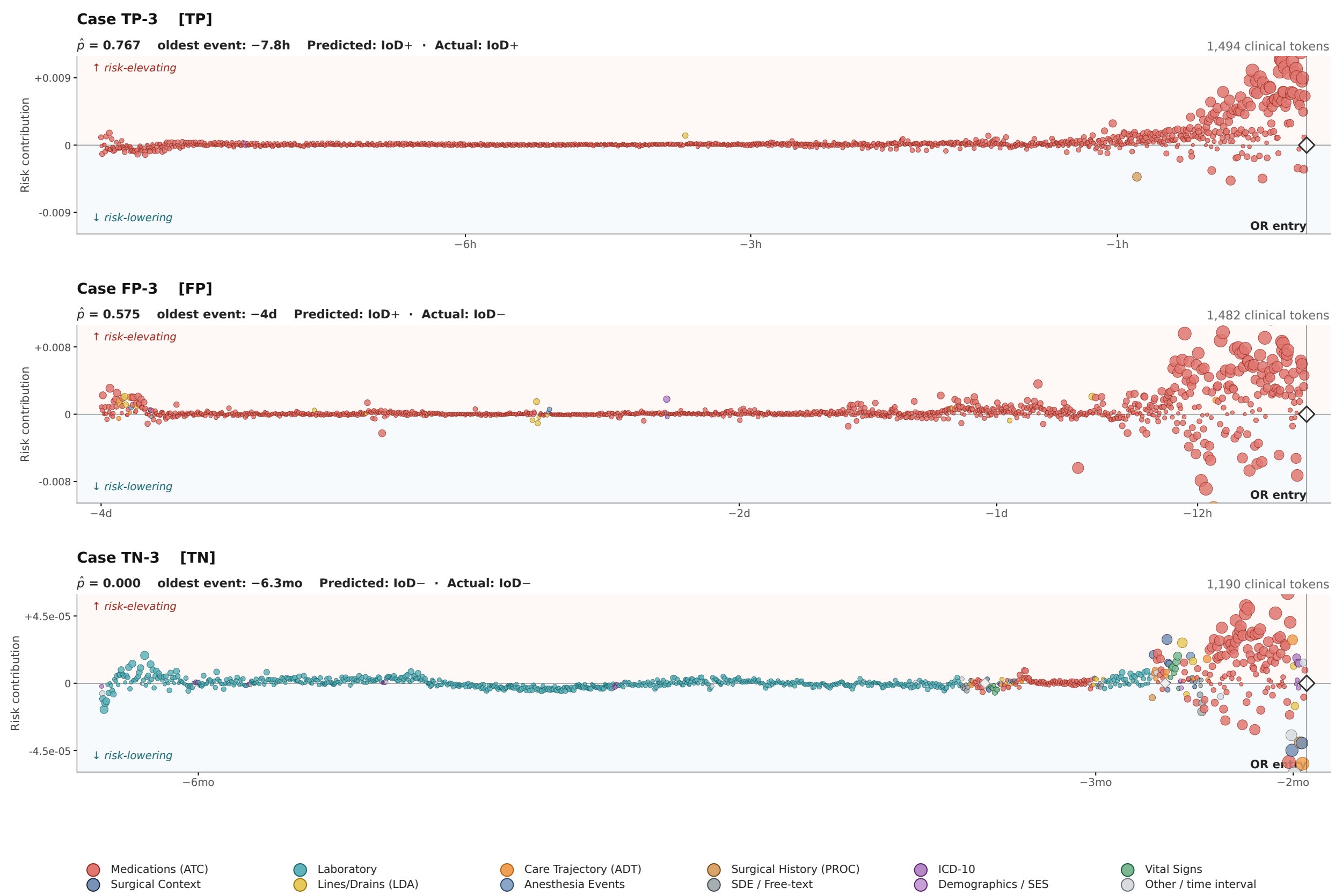


Figure S7: Extended per-encounter IG attribution, dot view (page 3 of 3). Layout as in Supplementary Fig. S5.

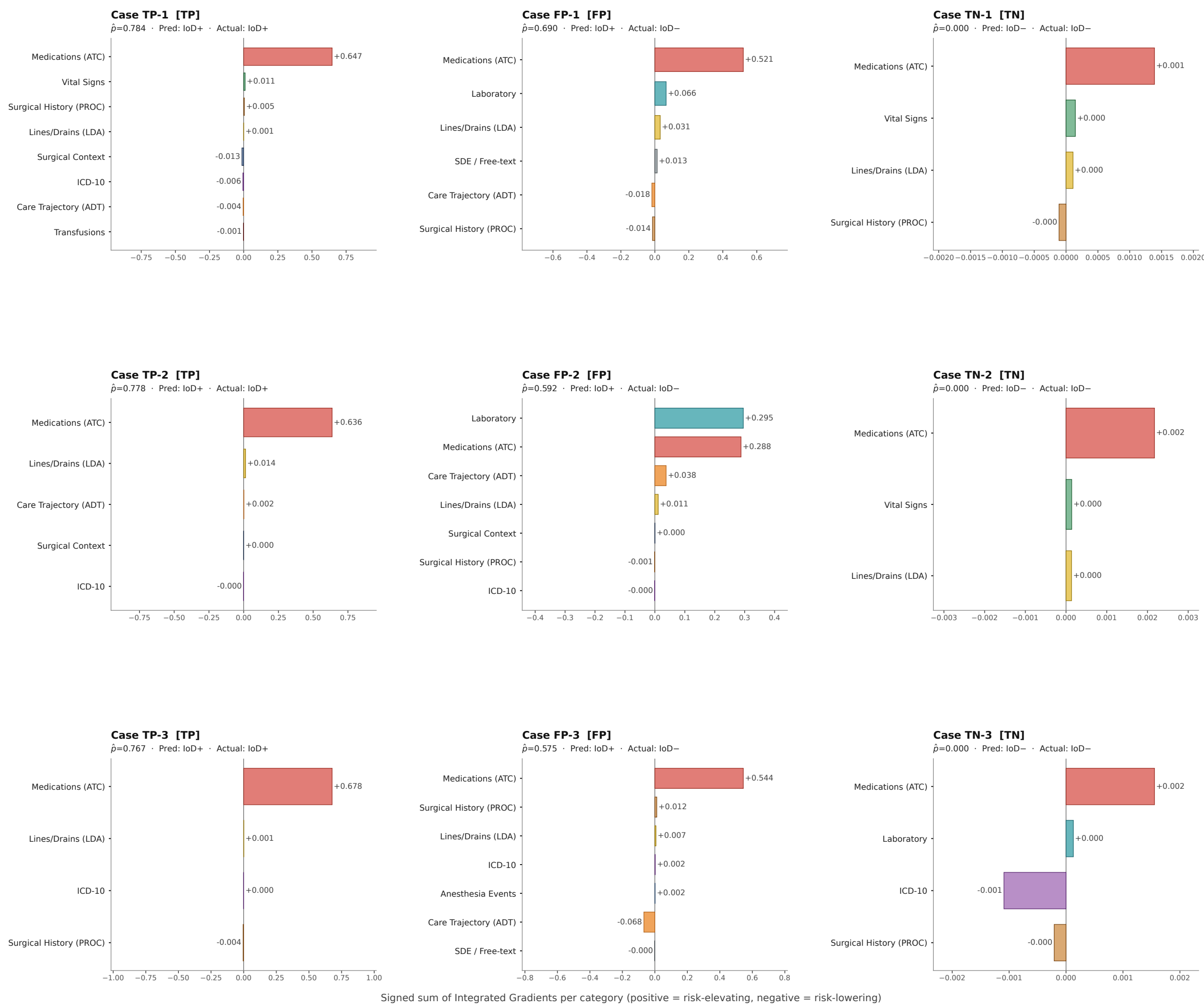


Figure S8: Per-patient category aggregation for the same nine cases as Supplementary Figures S5–S7. For each patient, every per-token IG is summed within its vocabulary category to produce a signed per-category contribution; positive bars (right) are net risk-elevating and negative bars (left) are net risk-lowering. Categories are sorted within each panel by absolute magnitude. Header reports the case label (TP/FP/TN-{1–3} at the Youden's $J$ operating point), $\hat{p}$, predicted and actual outcome.

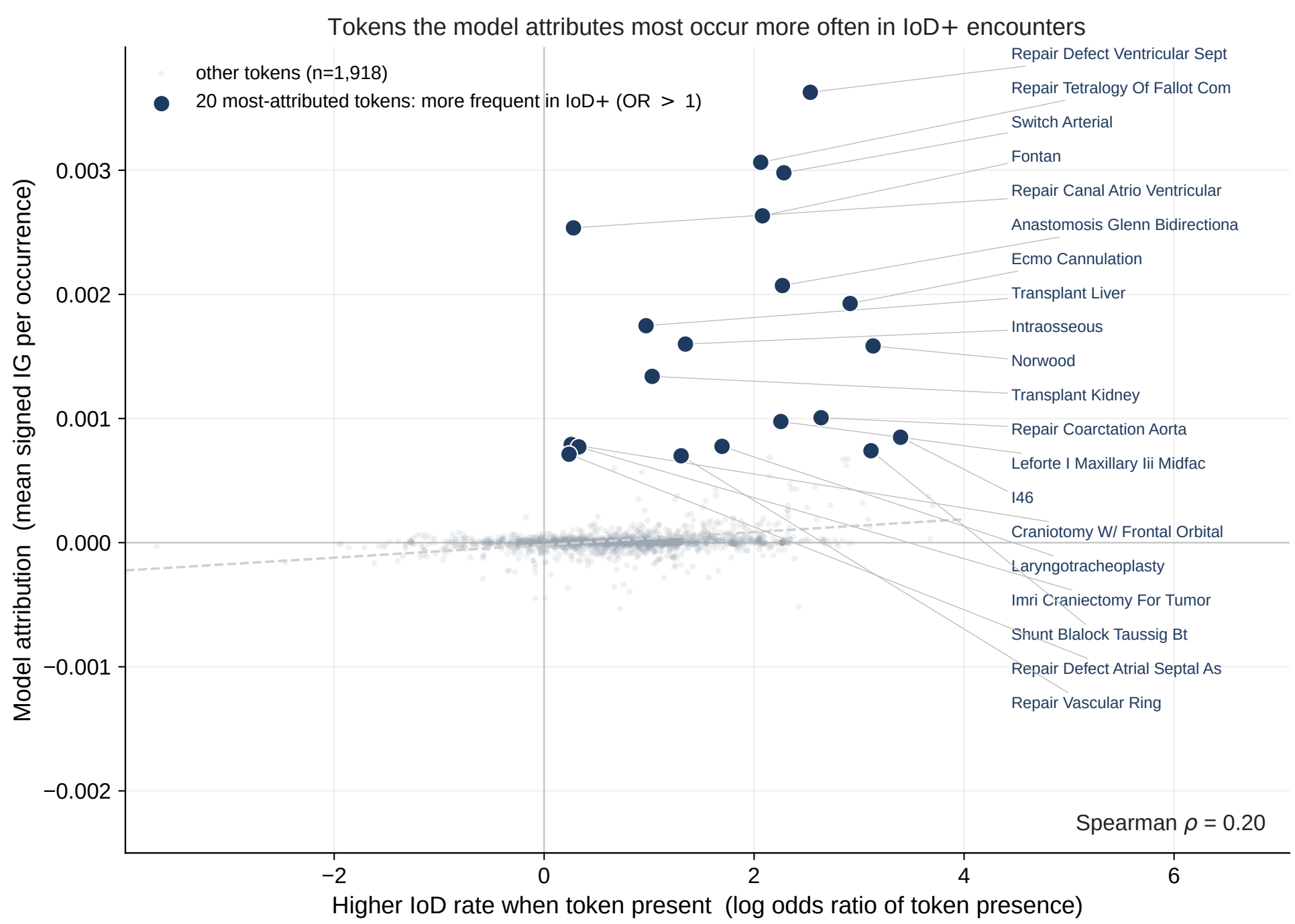


Figure S9: Attribution–association concordance. Each point is one token type occurring in ≥50 test encounters ($n$=1,938); the $x$-axis is the empirical IoD association (Haldane-corrected log odds ratio of token presence) and the $y$-axis is the model's mean signed Integrated-Gradients attribution per occurrence. The 20 most-attributed tokens are highlighted; all 20 have odds ratio $> 1$ (more frequent in IoD+ encounters). Attribution reflects marginal association, not causal effect or clinical utility.

## S7 Cohort Flow Diagram

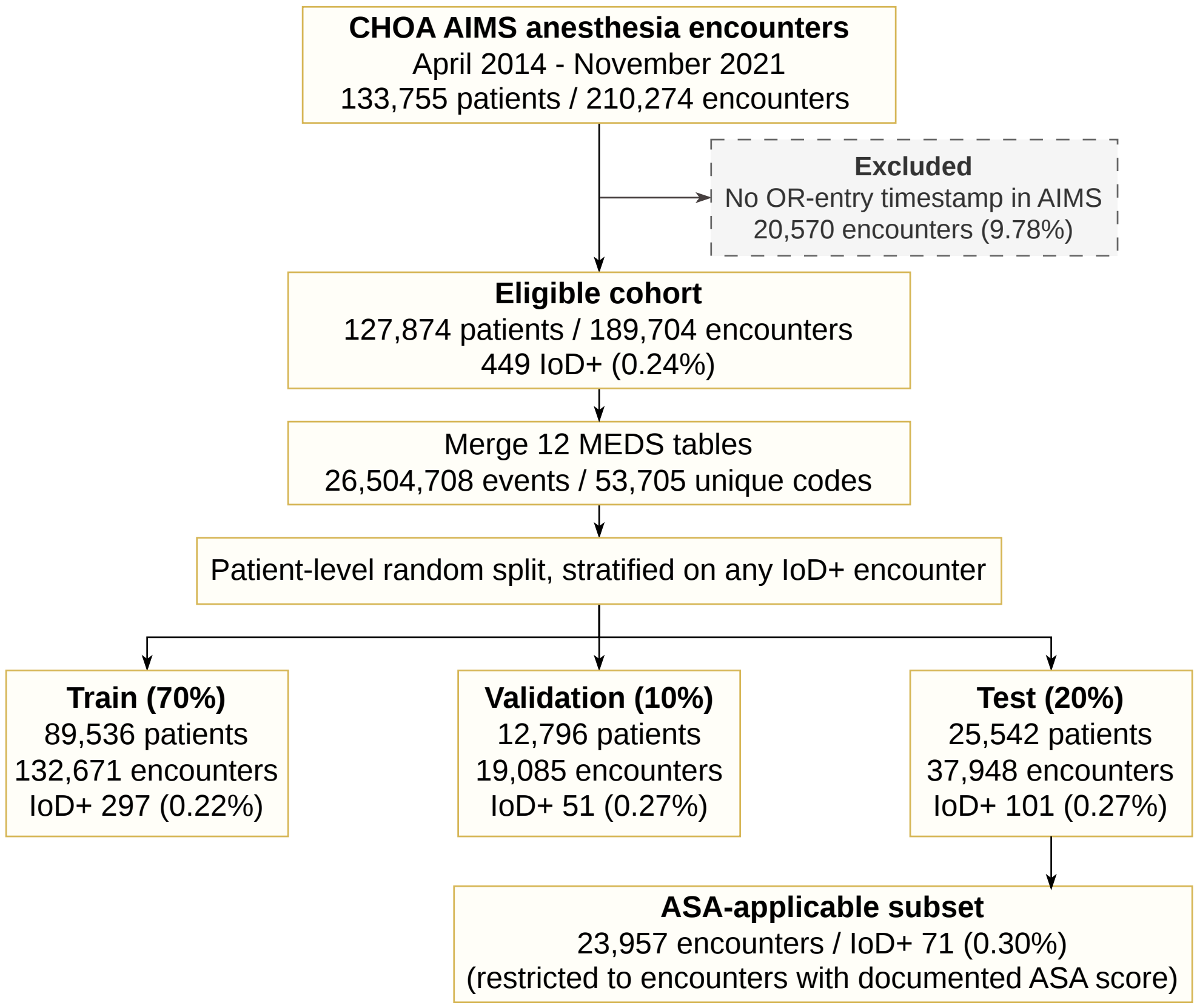


Figure S10: Cohort flow. CHOA AIMS anesthesia-encounter cohort (189,704 encounters, 127,874 patients) → 12 MEDS tables; patient-level random split stratified on any IoD+ encounter. The ASA-only baseline is further restricted to encounters with an ASA score recorded at the OR-entry timestamp (test $n$=23,957); subgroup analyses (Supplementary Table S15) use the broader set with any documented ASA score for that encounter (test $n$=37,388).

## S8 Supplementary Tables and Figures

Table S5: Data sources integrated into the MEDS pipeline. Patients = cohort patients with at least one event in the source; Events = events contributed by those cohort patients.

| **Source** | **Patients** | **Events** | **Notes** |
|---|---|---|---|
| Laboratory results | 8,100 | 3,212,503 | 6,695 codes, 84.9% numeric |
| Medical history | 91,218 | 468,202 | ICD-10 coded |
| Surgical history | 127,759 | 434,150 | 2,451 codes |
| Structured data elements | 74,427 | 1,004,400 | |
| Cardiology encounters | 16,565 | 66,052 | |
| ADT records | 127,790 | 2,030,173 | 54 departments |
| Medications | 69,822 | 9,783,362 | 2,690 drug codes |
| Nursing assessments (LDA) | 127,864 | 2,177,068 | 40 device types |
| Anesthesia patients | 127,873 | 4,085,524 | Timing, vitals, ASA, procedure |
| Anesthesia events | 127,867 | 594,092 | CPR, Quick Notes, etc. |
| Problem list | 107,276 | 1,025,151 | Active diagnoses |
| Transfusions | 11,020 | 196,669 | RBC, Plt, FFP, Cryoprecipitate |

Table S6: Vocabulary composition (6,170 tokens). "+ OTHER" = single catch-all bin within that category for codes below the top-$N$ cutoff. Sentinel tokens carry no clinical content and only control trajectory generation.

| **Category** | **Tokens** | **Notes** |
|---|---|---|
| *Coded clinical events* | | |
| ICD-10-CM (hierarchical) | 3,216 | 3-level decomposition |
| Encounter / surgical context | 520 | Procedure, ASA, admission type, OR markers |
| Laboratory (LAB) | 501 | Top-500 + OTHER |
| Surgical history (PROC) | 500 | Top-499 + OTHER |
| Structured data (SDE) | 301 | Top-300 + OTHER |
| Medications (ATC) | 277 | Hierarchical L1–L5 |
| Active problem list | 272 | Free-text names + END markers |
| Medical history | 248 | Free-text items + END markers |
| ADT transfers | 106 | 54 departments, in/out |
| Lines and drains (LDA) | 80 | Place/remove pairs |
| Transfusions | 14 | RBC, platelets, FFP, cryo, ... |
| Anesthesia events | 12 | CPR, Quick Note, labs taken, ... |
| Vital signs (preprocedure) | 8 | HR, SBP, DBP, $SpO_2$, RR, temp, ht, wt |
| *Patient context* | | |
| Demographics | 14 | Race, sex, ethnicity (atomic) |
| Home county | 51 | Top-50 GA + OTHER |
| Language | 6 | Top-5 + OTHER |
| Insurance | 6 | 4 named + OTHER + Unknown |
| Point of origin | 6 | 5 named + OTHER |
| *Numeric encoding and time* | | |
| Numeric quantiles | 10 | $Q_1$–$Q_{10}$, shared across codes |
| Time intervals | 19 | 5 min–>6 mo |
| *Sentinel control tokens* | | |
| Sentinels | 3 | `TIMELINE_END`, `OUTCOME//DEATH`, `MED//UNMAPPED` |
| **Total** | **6,170** | |

Table S7: Worked example of MEDS tokenization for a single elective cystoscopy encounter (anonymized patient). Top: raw MEDS rows recorded around the OR-entry timestamp (one row per clinical event). Bottom: the same segment after the four tokenization transformations applied (each numeric event paired with a quantile token $Q_k$, $k \in \{1, \ldots, 10\}$, computed from the per-code training-set decile boundaries; categorical tokens passed through unchanged). PORT reads the IoD probability off the hidden state at the trailing `ENCOUNTER//AN//OR_ENTRY` position.

| **Time** | **Code** | **Numeric value** |
|---|---|---|
| *Raw MEDS rows* | | |
| 2017-09-05 10:52 | `ENCOUNTER//AN//PRIMARY_PROCEDURE//CYSTOSCOPY` | – |
| 2017-09-05 10:52 | `ENCOUNTER//AN//ASA_SCORE` | 2 |
| 2017-09-05 10:52 | `VITAL//PREPROCEDURE//HEART_RATE` | 76 |
| 2017-09-05 10:52 | `VITAL//PREPROCEDURE//SBP` | 127 |
| 2017-09-05 10:52 | `VITAL//PREPROCEDURE//O2_SAT` | 100 |
| 2017-09-05 10:52 | `ENCOUNTER//AN//OR_ENTRY` | – |
| *Tokenized sequence (read left-to-right, top-to-bottom)* | | |
| `ENCOUNTER//AN//PRIMARY_PROCEDURE//CYSTOSCOPY` | | |
| `ENCOUNTER//AN//ASA_SCORE` $Q_3$ | | |
| `VITAL//PREPROCEDURE//HEART_RATE` $Q_4$ | | |
| `VITAL//PREPROCEDURE//SBP` $Q_6$ | | |
| `VITAL//PREPROCEDURE//O2_SAT` $Q_{10}$ | | |
| `ENCOUNTER//AN//OR_ENTRY` | | |

Table S8: Dataset splits (patient-level random assignment, stratified on whether the patient had any IoD+ encounter; seed 42). All encounters from a given patient are assigned to the same split to prevent patient-level leakage.

| **Split** | **Patients** | **Encounters** | **IoD+** | **IoD rate** |
|---|---|---|---|---|
| Train | 89,536 | 132,671 | 297 | 0.22% |
| Val | 12,796 | 19,085 | 51 | 0.27% |
| Test | 25,542 | 37,948 | 101 | 0.27% |
| **Total** | 127,874 | 189,704 | 449 | 0.24% |

Table S9: Sensitivity of test-set IoD prediction performance to within-patient encounter inclusion. "First-only" restricts to each patient's earliest test encounter ($n$=25,542, 73 IoD+); "No prior IoD" excludes the 133 encounters whose patient already had an IoD+ event ($n$=37,815, 95 IoD+). Full-cohort numbers ($n$=37,948, 101 IoD+) are reported in Table 1. AUROC and AUPRC with patient-clustered bootstrap 95% CIs (1,000 resamples). **Bold** = best within each subset. The ASA-only baseline is restricted to encounters with a documented ASA score (First-only $n$=11,580, 43 IoD+; No-prior $n$=23,824, 65 IoD+).

| | **First-only** | | **No prior IoD** | |
|---|---|---|---|---|
| **Model** | **AUROC ↑** | **AUPRC ↑** | **AUROC ↑** | **AUPRC ↑** |
| ASA score | 0.904 [.866, .938] | 0.046 [.021, .092] | 0.825 [.780, .867] | 0.023 [.012, .057] |
| LR (manual) | 0.806 [.758, .854] | 0.052 [.023, .105] | 0.767 [.722, .817] | 0.035 [.014, .079] |
| XGBoost (manual) | 0.791 [.740, .837] | 0.030 [.016, .061] | 0.749 [.699, .796] | 0.020 [.011, .044] |
| LR (MEDS) | 0.892 [.848, .928] | 0.055 [.033, .095] | 0.828 [.780, .874] | 0.031 [.018, .059] |
| XGBoost (MEDS) | 0.917 [.887, .945] | 0.133 [.079, .226] | 0.869 [.832, .904] | 0.097 [.053, .173] |
| BiLSTM (MEDS sequences) | 0.939 [.912, .962] | 0.256 [.158, .365] | 0.903 [.869, .932] | 0.192 [.112, .282] |
| Backbone, zero-shot | 0.555 [.468, .635] | 0.098 [.035, .171] | 0.589 [.517, .659] | 0.060 [.021, .122] |
| Backbone + linear probe | 0.882 [.833, .926] | 0.143 [.070, .232] | 0.794 [.745, .838] | 0.093 [.043, .161] |
| **PORT (Backbone + LoRA)** | **0.950 [.919, .975]** | **0.306 [.195, .408]** | **0.940 [.912, .965]** | **0.247 [.162, .344]** |

Table S10: PORT configuration and training hyperparameters. Top: generative backbone (decoder-only GPT-2 in the ETHOS style, trained from random initialization on pediatric perioperative MEDS timelines). Bottom: supervised LoRA fine-tuning for the IoD task on top of the frozen backbone.

| **Hyperparameter** | **Value** |
|---|---|
| *Generative backbone* | |
| Architecture | Decoder-only GPT-2 (causal LM) |
| Parameters | 47.25M |
| Vocabulary size | 6,208 (6,170 rounded to nearest 64) |
| Layers | 6 |
| Attention heads | 12 |
| Hidden dimension | 768 |
| Context length | 2,048 tokens |
| Dropout | 0.3 (residual, attention, embedding) |
| Activation | GELU |
| Optimizer | AdamW ($\beta_1$=0.9, $\beta_2$=0.95, wd= 0.1) |
| Peak learning rate | $6 \times 10^{-4}$ (cosine decay) |
| Min learning rate | $1 \times 10^{-5}$ |
| Warmup steps | 2,000 |
| Total steps | 200,000 |
| Batch size | 32 / GPU $\times$ 8 GPUs = 256 |
| Gradient accum. | 16 steps (effective: 2 per worker) |
| Effective batch | $\sim$0.5M tokens/step |
| Hardware | 8 $\times$ NVIDIA H200 (143 GB each) |
| Training time | $\sim$17 hours |
| *LoRA fine-tuning (IoD task)* | |
| LoRA target | QKV projection (`c_attn`, all 6 layers) |
| Rank $r$ | 8 |
| Scaling $\alpha$ | 16 |
| Adapter dropout | 0.1 |
| LoRA trainable params | 147,456 (0.31% of backbone) |
| Classification head | 2-layer MLP (768 $\to$ 256 $\to$ 1), ReLU |
| Head trainable params | 197,121 (0.42% of backbone) |
| Total trainable | 344,577 (0.73% of backbone) |
| Loss function | Unweighted binary cross-entropy (BCE) |
| Optimizer | AdamW (wd = 0.01) |
| Head learning rate | $10^{-3}$ |
| Adapter learning rate | $10^{-4}$ |
| Batch size | 64 |
| Max epochs | 15 (typical convergence 5–10) |
| Early stopping | Patience-5 on validation AUROC |
| Checkpoint selection | Best validation AUROC |
| Random seeds | 42, 123, 456 (3 independent fits) |

Table S11: IoD stop tokens used during zero-shot inference. In the hierarchical ATC vocabulary, vasoactive drugs are identified by a combination of parent-class and leaf-suffix tokens.

| ATC Token | Drug | IoD Component |
|---|---|---|
| `AN_EVENT//CPR` | – | Cardiac arrest (IoD1) |
| `ATC//C01` | Cardiac stimulants (class) | IoD3/4 |
| `ATC//SFX//A24` | Epinephrine | IoD3/4 |
| `ATC//SFX//A04` | Dopamine | IoD4 |
| `ATC//SFX//E02` | Milrinone | IoD4 |
| `ATC//SFX//A03` | Norepinephrine | IoD4 |
| `ATC//SFX//A06` | Phenylephrine | IoD3/4 |
| `ATC//SFX//A01` | Vasopressin | IoD3/4 |
| `ATC//SFX//B10` | Adenosine | IoD3 |
| `ATC//SFX//A26` | Ephedrine | IoD3 |
| `LDA//ARTERIAL_LIN` | Arterial line | IoD5 |

Table S12: Baseline hyperparameter sweep configurations and validation AUPRC. All baselines were re-tuned under the unweighted-loss regime ($w_+ = 1$ for LR/BiLSTM, `scale_pos_weight` $= 1$ for XGBoost) so that loss configuration matches PORT. LR was tuned by 6-point grid search over $C \in \{10^{-3}, \ldots, 10^2\}$. XGBoost was tuned by 50-trial random search over trees, depth, learning rate, subsample, column-sample, min child weight, and $\lambda$ (only the best-performing combination is shown; remaining hyperparameters at common defaults). BiLSTM was tuned over a 6-configuration grid (hidden dim, layers, dropout, learning rate; vocabulary 2,000 most-frequent codes, sequence length 512, embedding dim 64 fixed). The best configuration per model (**bold**) was selected by validation AUPRC and evaluated on the held-out test set; the corresponding test metrics are reported in the main Table 1.

| Model | Configuration | Val AUPRC |
|---|---|---|
| LR (manual) | $C = 0.1$ | **0.033** |
| LR (MEDS) | $C = 0.001$ | **0.018** |
| XGB (manual) | 200 trees, depth 8, lr 0.05 | **0.028** |
| XGB (MEDS) | 1000 trees, depth 5, lr 0.03 | **0.077** |
| BiLSTM | hidden 128, 2 layers, dropout 0.3, lr 1e-3 | **0.258** |
| BiLSTM | hidden 256, 2 layers, dropout 0.5, lr 1e-3 | 0.254 |
| BiLSTM | hidden 256, 2 layers, dropout 0.1, lr 1e-3 | 0.244 |
| BiLSTM | hidden 256, 3 layers, dropout 0.3, lr 1e-3 | 0.226 |
| BiLSTM | hidden 512, 2 layers, dropout 0.3, lr 1e-3 | 0.223 |
| BiLSTM | hidden 256, 2 layers, dropout 0.3, lr 1e-3 | 0.156 |

Table S13: Occlusion analysis: AUROC and AUPRC when each token category is masked. Baseline (no masking): AUROC = 0.942, AUPRC = 0.237. Rows sorted by AUROC drop. Category vocabulary sizes are in Supplementary Table S6.

| **Category** | **AUROC ↑** | **ΔAUROC** | **AUPRC ↑** | **ΔAUPRC** |
|---|---|---|---|---|
| Surgical Context (ENCOUNTER) | 0.885 | −0.056 | 0.209 | −0.029 |
| Surgical History (PROCEDURE) | 0.896 | −0.045 | 0.230 | −0.007 |
| ICD-10 Codes | 0.922 | −0.020 | 0.235 | −0.002 |
| Medications (ATC) | 0.928 | −0.014 | 0.048 | −0.189 |
| Care Trajectory (ADT) | 0.931 | −0.011 | 0.237 | −0.000 |
| Point of Origin | 0.941 | −0.001 | 0.230 | −0.007 |
| Structured Data (SDE) | 0.941 | −0.001 | 0.236 | −0.001 |
| Anesthesia Events | 0.941 | −0.001 | 0.238 | +0.001 |
| Laboratory | 0.941 | −0.001 | 0.243 | +0.006 |
| Vital Signs | 0.941 | −0.001 | 0.239 | +0.002 |
| Language | 0.941 | −0.000 | 0.229 | −0.008 |
| Problem List (free-text) | 0.942 | −0.000 | 0.238 | +0.000 |
| Insurance | 0.942 | −0.000 | 0.235 | −0.002 |
| Home County | 0.942 | +0.000 | 0.237 | +0.000 |
| Demographics | 0.942 | +0.000 | 0.237 | +0.000 |
| Medical History (free-text) | 0.942 | +0.000 | 0.237 | −0.000 |
| Transfusions | 0.942 | +0.000 | 0.238 | +0.001 |
| Lines/Drains (LDA) | 0.942 | +0.000 | 0.239 | +0.002 |

Table S14: IoD prevalence and encounter counts by ASA physical status class ($N$=186,954 analysis-cohort encounters with a recorded ASA score; 2,750 without). Aggregate rows (≥III, ≥IV) include 94 ASA VI encounters (brain-dead organ donors; 18 IoD+, 19.1%) not shown individually.

| **ASA** | **Encounters** | **IoD+** | **IoD−** | **Prevalence** |
|---|---|---|---|---|
| I | 46,064 | 4 | 46,060 | 0.01% |
| II | 78,953 | 46 | 78,907 | 0.06% |
| III | 56,651 | 198 | 56,453 | 0.35% |
| IV | 5,038 | 159 | 4,879 | 3.16% |
| V | 154 | 21 | 133 | 13.64% |
| I–II | 125,017 | 50 | 124,967 | 0.04% |
| ≥III | 61,937 | 396 | 61,541 | 0.64% |
| ≥IV | 5,286 | 198 | 5,088 | 3.75% |

Table S15: PORT performance by ASA subgroup on the test set ($n$=37,388 with an ASA class documented at any time; this differs from the 23,957 with a pre-operatively available ASA used as the ASA-only predictor in Table 1). PPV is computed at each subgroup's own Youden's $J$ threshold (the discrimination-optimal operating point within that subgroup).

| **Subgroup** | $n$ | **IoD+** | **Prev.** | **AUROC ↑** | **AUPRC ↑** | **PPV ↑** |
|---|---|---|---|---|---|---|
| All | 37,388 | 101 | 0.27% | 0.942 | 0.238 | 1.9% |
| ASA $\geq$ 3 | 12,308 | 89 | 0.72% | **0.931** | 0.274 | 3.0% |
| ASA $\geq$ 4 | 987 | 46 | 4.66% | 0.872 | **0.471** | **11.9%** |
| ASA I | 9,236 | 0 | 0.00% | — | — | — |
| ASA II | 15,844 | 12 | 0.08% | 0.783 | 0.010 | 0.5% |
| ASA III | 11,321 | 43 | 0.38% | 0.909 | 0.061 | 1.7% |
| ASA IV | 939 | 35 | 3.73% | 0.845 | 0.333 | 9.9% |

Table S16: Per-encounter inference cost on a single GPU (batch size 1, mean ± std over 495 test encounters; 5 warmup encounters discarded). The GPU-memory column reports the model's resident memory during inference; a complete production deployment additionally requires the standard CUDA / framework runtime overhead and the MEDS preprocessing pipeline.

| **Model** | **Params** | **Latency (ms / encounter)** | **Peak GPU memory (MB)** |
|---|---|---|---|
| PORT (LoRA) | 47.25M | 7.19 ± 0.08 | 318 |
| BiLSTM (tuned) | 0.73M | 12.68 ± 0.23 | 81 |

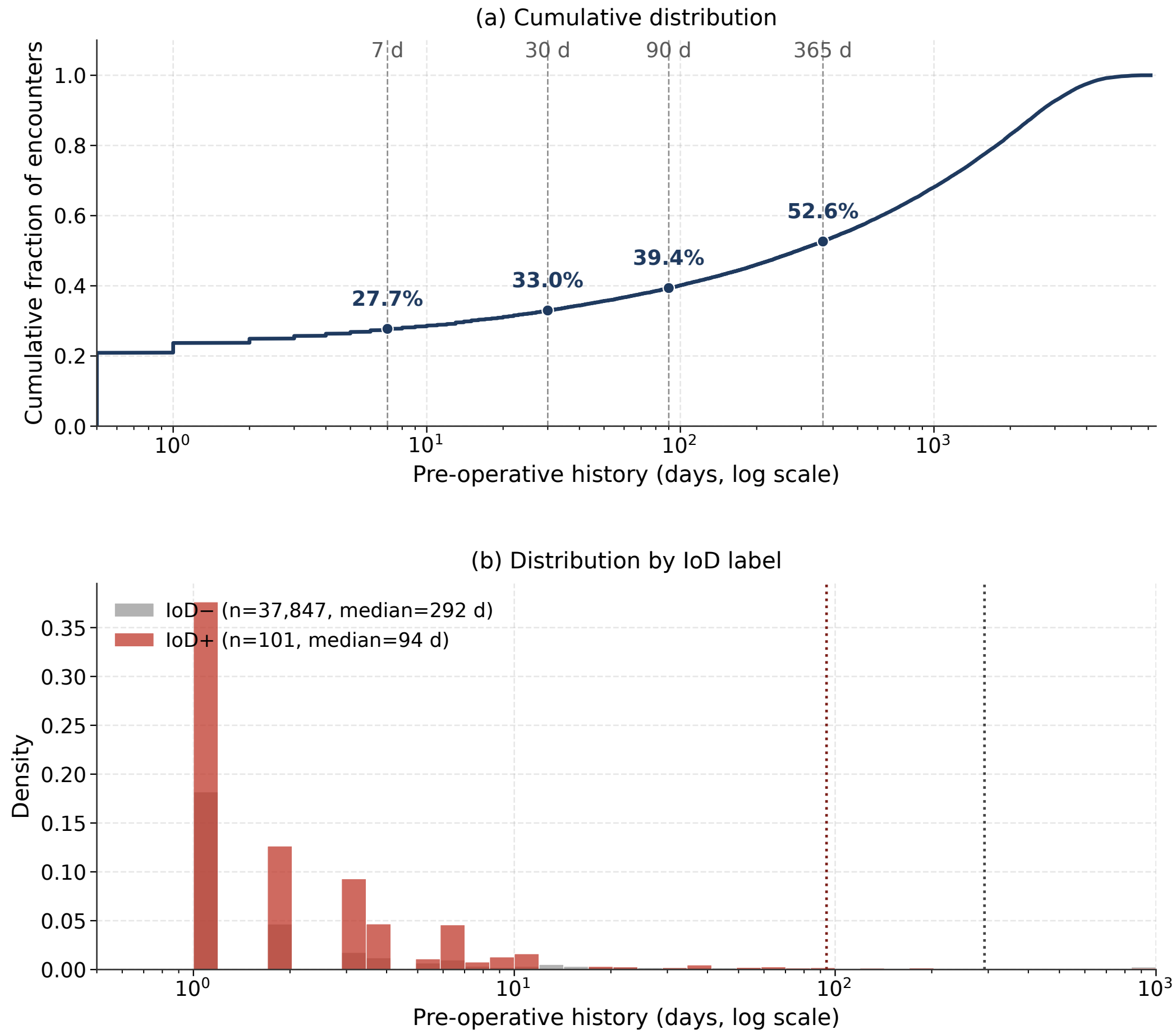


Figure S11: Pre-operative history length distribution on the held-out test set ($n$=37,948, 101 IoD+). (a) Cumulative distribution on a log day axis, with the 7/30/90/365-day window cutoffs marked and the cumulative fraction of encounters reached at each. (b) Density by IoD label: IoD+ encounters have a shorter median history (94 d) than IoD− encounters (292 d).

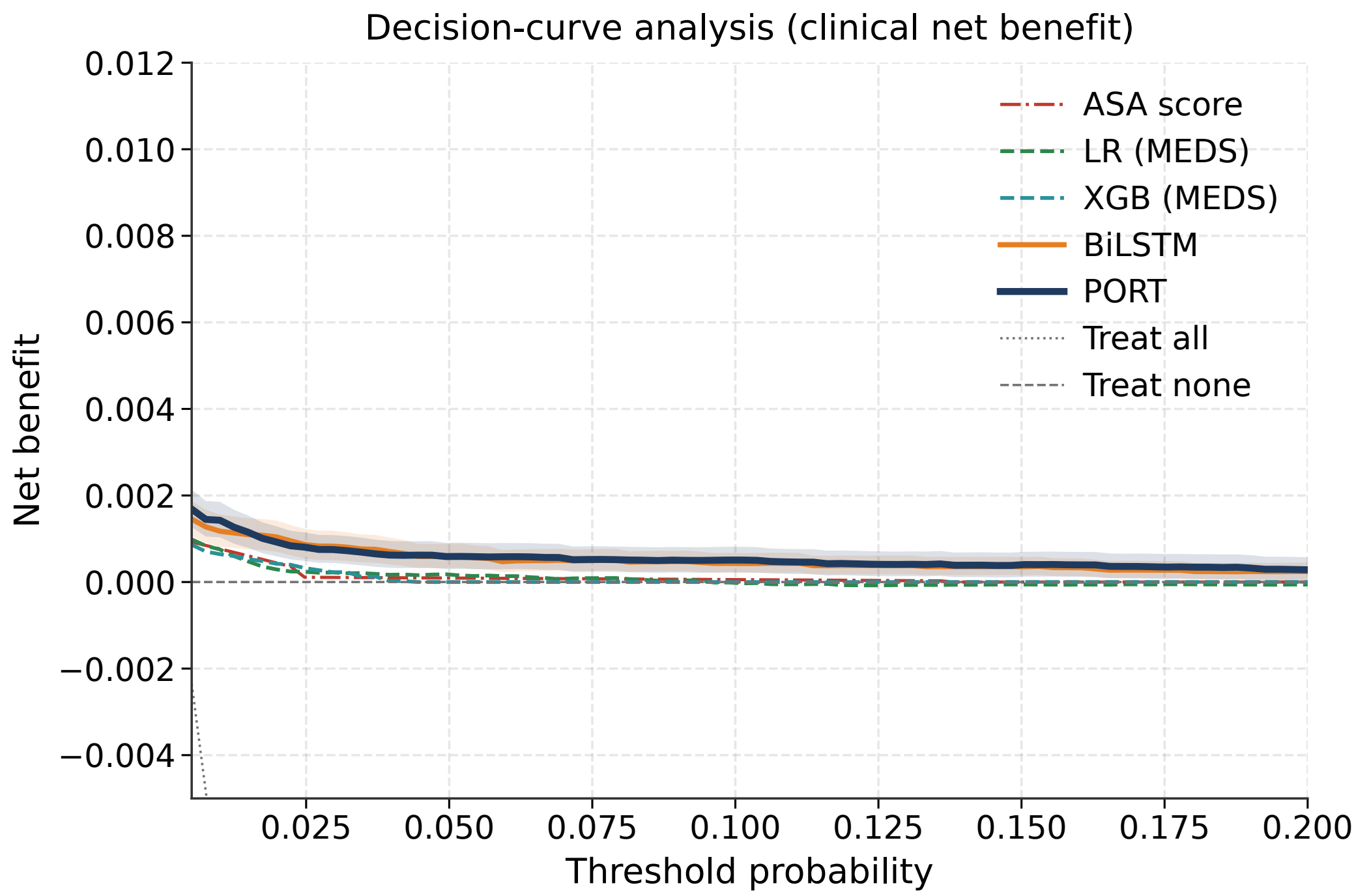


Figure S12: Decision-curve analysis for IoD prediction across clinically realistic threshold probabilities (0.5–20%). Net benefit is computed as $\mathrm{TP}/N-(\mathrm{FP}/N)\cdot t/(1-t)$, where $t$ is the threshold probability used to flag a patient as high-risk. PORT yields the largest net benefit over the full threshold range and dominates "treat-all" and "treat-none" strategies as well as every supervised baseline.

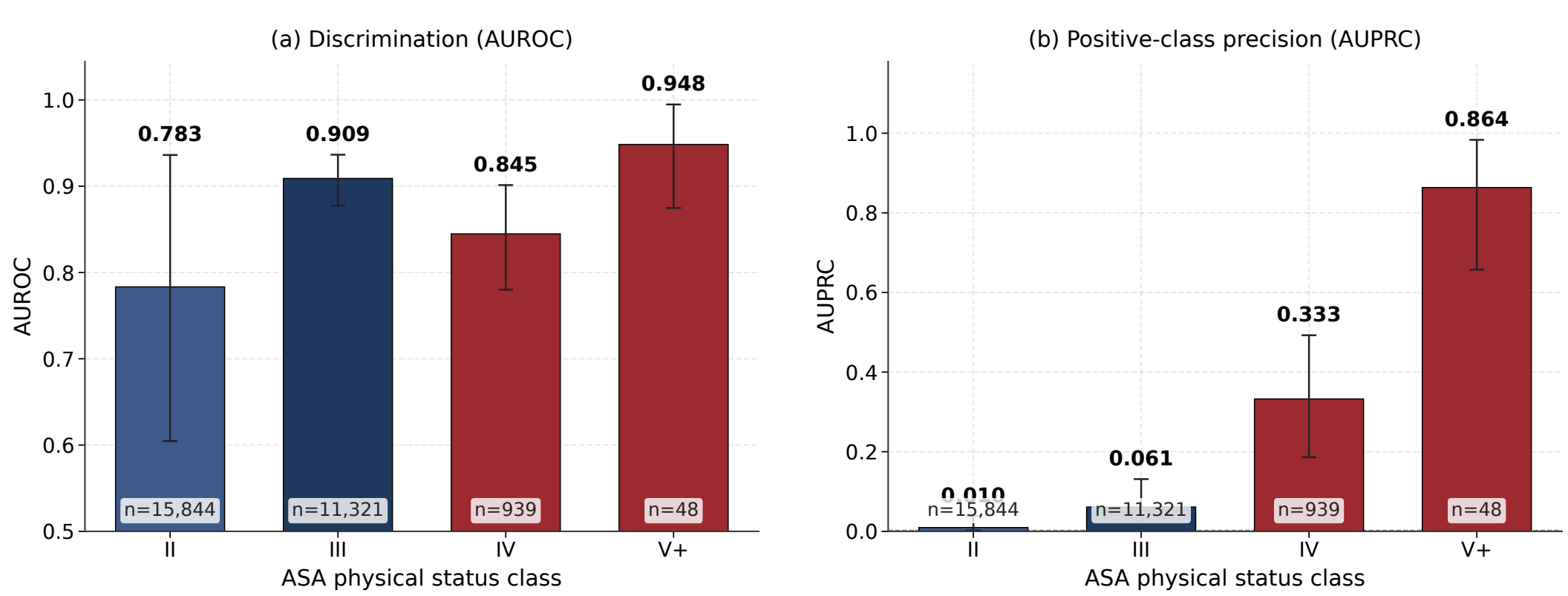


Figure S13: PORT performance by ASA physical status class on the held-out test set ($n$=37,388). (a) AUROC and (b) AUPRC per class with bootstrap 95% CIs and sample sizes; V+ aggregates V and VI.

## S9 TRIPOD+AI Reporting Checklist

This study is a prediction-model *development* study with internal (patient-level held-out) validation and a temporal (calendar-split) validation; geographic-external and prospective validation are designated future work. We report adherence to the TRIPOD+AI 2024 statement.[59] Section and table references below point to the main text unless prefixed "S" (Supplementary). "N/A" denotes items not applicable to a single-institution development study with no geographic-external or prospective evaluation, no model updating, and no multi-cluster analysis.

Table S17: TRIPOD+AI (2024) 27-item reporting checklist mapped to the location in this manuscript where each item is addressed. "Dev." = model development; "eval." = internal evaluation on the held-out test set. This is a development study with internal and temporal validation.

| Item | Checklist item (abbreviated) | Where addressed |
|---|---|---|
| **Title and Abstract** | | |
| 1 | Identify the study as developing/evaluating a multivariable prediction model, the target population, and the predicted outcome. | Title; Abstract. |
| 2 | Provide a structured summary following the TRIPOD+AI for Abstracts checklist. | Abstract (unstructured; incorporates TRIPOD+AI for Abstracts elements). |
| **Introduction** | | |
| 3a | Healthcare context and rationale, with reference to existing models. | Introduction (ASA, RACHS-1, STS-CHSD, FLEX, ETHOS). |
| 3b | Target population and intended use within the care pathway, including intended users. | Background ("This work"); Objectives; Discussion ("Clinical utility and deployment"). |
| 3c | Known health inequalities between sociodemographic groups. | Methods, "Study cohort" (safety-net population) and SES tokens; Results, occlusion (Point of Origin / Language); Table 3 (race/ethnicity by outcome). |
| 4 | Study objectives, stating development and/or validation. | Introduction, final paragraph (develop and internally evaluate PORT). |
| **Methods** | | |
| 5a | Sources of data (dev. and eval.), rationale, representativeness. | Methods, "Study cohort and data sources"; Supplementary Table S5; Supplementary Fig. S10. |
| 5b | Dates of participant accrual / follow-up. | Methods, "Study cohort" (AIMS encounters Apr 2014–Nov 2021; structured EHR Mar 2007–Oct 2022). |
| 6a | Study setting, number and location of centres. | Methods, "Study cohort" (two CHOA campuses, Egleston and Scottish Rite). |
| 6b | Eligibility criteria for participants. | Methods, "Study cohort" (all AIMS anesthesia encounters; exclusion of encounters lacking OR-entry timestamp); Table 4 note; Supplementary Fig. S10. |

*continued on next page*

| Item | Checklist item (abbreviated) | Where addressed |
|---|---|---|
| 6c | Treatments received and how handled. | Methods, "Dataset splits" (per-encounter; prior surgeries retained as pre-op information); cardiac-surgery encounters retained and handled by stratification (Results; Supp. Sec. S1). |
| 7 | Data pre-processing and quality checking, including across sociodemographic groups. | Methods, "MEDS tokenization"; Section 4.2 (MEDS harmonization, ICD/ATC decomposition, frequency cutoffs, atomic demographic decomposition). |
| 8a | Outcome definition, time horizon, how/when assessed, rationale, consistency across groups. | Methods, "IoD label definition"; Table 4; Section 4.3 (structured-EHR-derived components apply uniformly across encounters). |
| 8b | Qualifications/demographics of outcome assessors (subjective interpretation). | Methods, "IoD label definition" (components selected/refined by pediatric anesthesiologists); Supp. Sec. 4.3 (IoD2 keyword list developed with a board-certified pediatric anesthesiologist). Assessor demographics not collected. |
| 8c | Blinding of outcome assessment. | Partially: IoD1/IoD3–IoD5 are deterministic structured-data rules (no subjective scoring); IoD2 audit was performed without access to model predictions. Formal blinding protocol N/A (rule-based labeling). |
| 9a | Choice of initial predictors and any pre-selection. | Methods, "MEDS tokenization" and "Backbone" (all structured pre-OR events tokenized; no manual predictor pre-selection for PORT); baseline feature sets in "Baseline models". |
| 9b | Definition of all predictors, how/when measured. | Methods, "MEDS tokenization"; Section 4.2; Supplementary Table S6; Supplementary Table S7; cutoff at In OR timestamp. |
| 9c | Qualifications/demographics of predictor assessors (subjective interpretation). | N/A: predictors are structured EHR events recorded in routine care; no subjective predictor interpretation. ATC mapping (objective ontology) reviewed by authors (Section 4.2). |
| 10 | How study size was determined; justification of sufficiency. | Methods, "Dataset splits" (full available cohort used; counts in Supplementary Table S8); Discussion / Supp. Sec. S3 (data-efficiency ablation characterizing sufficiency). No a priori power calculation (all eligible encounters used). |

*continued on next page*

| Item | Checklist item (abbreviated) | Where addressed |
|---|---|---|
| 11 | Handling of missing data; reasons for omitting data. | Methods, "Study cohort" (encounters without OR-entry timestamp excluded); Methods, "MEDS tokenization" (per-category `OTHER` fallback; absent events simply not tokenized in a sequence model); Table 3 note (gestational-age availability). |
| 12a | How data were used in analysis; partitioning. | Methods, "Dataset splits" (patient-level 70/10/20 train/val/test, stratified, leakage-controlled); Supplementary Table S8. |
| 12b | Handling of predictors (functional form, rescaling, transformation). | Methods, "MEDS tokenization" (numeric codes as code–quantile pairs; per-code decile boundaries; time-gap discretization). |
| 12c | Model type, rationale, all building steps, hyperparameter tuning, internal validation. | Methods, "Backbone architecture and pretraining", "Supervised adaptation", "Baseline models"; Supplementary Section S2 and Supplementary Table S3 (LoRA sweep); Supplementary Table S12; Supplementary Table S10; validation-AUROC checkpoint selection / early stopping. |
| 12d | Heterogeneity in parameters/performance across clusters. | N/A: single institution; no multi-cluster (hospital/country) structure. Within-cohort heterogeneity addressed via subgroup analyses (item 23a). |
| 12e | Measures and plots for model performance, and model comparison. | Methods, "Metrics and calibration" (AUROC, AUPRC, Brier, BSS, ECE, reliability, DCA, subgroup); rationale for AUPRC as primary metric. |
| 12f | Any model updating/recalibration from evaluation. | N/A: no post-hoc recalibration; calibration assessed as-is (Methods, "Metrics"; Results; Discussion notes "without auxiliary calibration"). |
| 12g | How predictions were computed for evaluation. | Methods, "Zero-shot IoD inference" and "Supervised adaptation" (OR-entry hidden-state readout, sigmoid head); equations (1)–(2); code repository (Data and code availability). |
| 13 | Class-imbalance methods and any subsequent recalibration. | Methods, "Supervised adaptation" and "Baseline models" (unweighted BCE selected; weighted BCE / focal / oversampling compared); Supplementary Section S2, Supplementary Table S3. |

*continued on next page*

| Item | Checklist item (abbreviated) | Where addressed |
|---|---|---|
| 14 | Approaches used to address model fairness, and rationale. | Partially addressed: performance reported across ASA-acuity and demographic context; SES tokens included; no observed-performance fairness disparity mitigation was performed (no reweighting/constraints). See Discussion / Limitations and Gaps list. |
| 15 | Model output; rationale for any classification thresholds. | Methods, "Zero-shot inference" / "Supervised adaptation" (probability output); Results, "Risk stratification" and Table 2 (sensitivity/specificity/Youden operating points). |
| 16 | Differences between development and evaluation data. | N/A: development and internal evaluation drawn from the same source via a patient-level split (no separate evaluation cohort); identical setting, eligibility, outcome, predictors. |
| 17 | Ethics approval and consent/waiver. | "Ethics approval" section (CHOA IRB STUDY00001114; Duke IRB Pro00116862; waiver of informed consent). |
| **Open Science** | | |
| 18a | Source of funding and role of funders. | "Funding" section (HeRO / Children's–Georgia Tech Cardiac Research Fund). |
| 18b | Conflicts of interest / financial disclosures. | "Competing interests" section (none declared). |
| 18c | Where the study protocol can be accessed. | Not prepared / not separately registered as a protocol. See Gaps list. |
| 18d | Study registration (register name and number). | Not registered (retrospective EHR development study). See Gaps list. |
| 18e | Availability of study data. | "Data and code availability" (PHI-restricted; de-identified timelines/weights on request under DUA). |
| 18f | Availability of analytical code. | "Data and code availability" (`https://github.com/Kamaleswaran-Lab/PORT`). |
| **Patient and Public Involvement** | | |
| 19 | Patient and public involvement. | No patient or public involvement in design, conduct, or reporting. See Gaps list. |
| **Results** | | |
| 20a | Flow of participants, with/without outcome; follow-up. | Supplementary Fig. S10 (cohort flow); Supplementary Table S8 (encounters and IoD+ per split); Table 4 note (exclusions). |
| 20b | Characteristics overall and by source/setting, incl. demographics, outcome events, missing data; differences across groups. | Table 3 (characteristics by IoD outcome with $p$-values); Supplementary Table S5; Supplementary Table S14. |

*continued on next page*

| Item | Checklist item (abbreviated) | Where addressed |
|---|---|---|
| 20c | For evaluation, compare predictor/outcome distribution with development data. | N/A: internal split of a single cohort; train/test drawn from the same distribution by construction (Supplementary Table S8). |
| 21 | Number of participants and outcome events in each analysis. | Supplementary Table S8 (dev/val/test); Table 1 caption (test $n$=37,948, 101 IoD+); Supplementary Table S10 (tuning seed/config). |
| 22 | Full model specification to allow prediction in new individuals. | Methods, "Supervised adaptation" (architecture, readout, equations); Supplementary Table S10 (full hyperparameters); code/weights via "Data and code availability". |
| 23a | Performance estimates with CIs, including key subgroups; plots. | Table 1 (AUROC/AUPRC with 95% CIs); Fig. 2; Fig. 3; Fig. 6; Supplementary Tables S15, S2, S9; Supplementary Figures S13, S12. |
| 23b | Heterogeneity in performance across clusters. | N/A (single institution). Subgroup heterogeneity (ASA, cardiac vs. noncardiac) reported as 23a: Supplementary Tables S15, S2. |
| 24 | Results of any model updating. | N/A: no model updating/recalibration performed. |
| **Discussion** | | |
| 25 | Overall interpretation, including fairness, vs. objectives and prior studies. | Discussion, "Why zero-shot…", "Comparison with existing tools…", "Interpreting perioperative risk signals"; fairness and equity are addressed under "Limitations" (characterized but not formally mitigated; see item 14). |
| 26 | Limitations and their effects on bias, uncertainty, generalisability. | Discussion, "Limitations" (single-institution, retrospective, IoD2 label noise, free-text exclusion, no geographic-external or prospective validation, optimism from recurring tokens, fairness not formally mitigated). |
| 27a | How poor-quality/unavailable input data should be handled at implementation. | Discussion, "Clinical utility and deployment" and "Limitations" (required OR-entry timestamp / ASA / procedure code; per-category `OTHER` fallback; 7-day-window robustness; documentation-timing/leakage caveat). |
| 27b | User interaction and required expertise. | Discussion, "Clinical utility and deployment" (automated per-encounter score; clinician-facing dashboard, Fig. 1; inference cost Supplementary Table S16). |

*continued on next page*

| Item | Checklist item (abbreviated) | Where addressed |
|---|---|---|
| 27c | Next steps for future research; applicability and generalisability. | Discussion, "Future work" (multi-modal/intraoperative extension, multi-outcome adapters, prospective and multi-site external validation). |

*Items not fully addressed (development-study scope):* no prospective protocol or registration (18c, 18d); no patient or public involvement (19); model fairness characterized descriptively but not formally mitigated (14); and no a priori sample-size calculation (10; all eligible encounters were used). These gaps are disclosed in the Ethics, Funding, and Data-and-code-availability statements and in the Discussion limitations.
